# Halide-Based Solid Electrolytes for Advanced All-Solid-State Batteries: Design, Interfaces, and Electrochemical Performance

**DOI:** 10.1007/s40820-026-02251-3

**Published:** 2026-06-22

**Authors:** Shivaraju Guddehalli Chandrappa, Gerardo Morell, Ram S. Katiyar

**Affiliations:** Department of Physics, University of Puerto Rico, San Juan, Puerto Rico, 00931 USA

**Keywords:** Lithium-ion batteries, Post-lithium-ion batteries, Halide-based solid electrolytes, Interfacial stability, All-solid-state batteries, High-voltage-based batteries

## Abstract

Halide-based solid electrolytes (HSEs) have garnered substantial interest for all-solid-state batteries (ASSBs) due to their wide electrochemical windows, moderate-to-high room-temperature ionic conductivity, and enhanced air stability over traditional sulfide and oxide-based SEs. This review consolidates recent advances in HSEs, focusing on the link between structure, compositions, and materials properties that influence the transport of lithium-ion (Li-ion) and post-lithium-ion (P-Li-ion) and their stability at the interface. Based on the chemistry of their central metal, HSEs are divided into five classes; key factors influencing ionic conductivity are examined. Nevertheless, despite these benefits, many challenges remain, including interfacial instability, the trade-off between ionic conductivity and electrochemical stability, mechanical challenges, and material costs. The main synthesis methods, mechanochemical, co-melting, and wet-chemical, are investigated for phase formation, scalability, and defect control. The link between synthesis, microstructure, and device-level performance metrics, including critical current density, area-specific resistance, and cycle life, is examined. The strategies, involving bilayer and dual-electrolyte design as well as interface engineering, are analyzed to reduce interfacial resistance and dendrite growth. The applications of HSE in Li-ion and P-Li-ion systems are examined. This review offers a detailed framework and delineates potential research paths to advance scalable, high-performance HSEs for next-generation ASSBs.

## Highlights

- General classification and methodology guidelines to halide-based solid electrolytes for high-voltage all-solid-state batteries.
- A comprehensive evaluation of synthesis and interfacial modification methods that direct the enhancement of ionic conductivity and electrochemical stability.
- Potential uses of halide-based solid electrolytes in full-cell configurations with cathodes based on high voltage in Li-ion batteries and beyond-Li-ion systems, such as Li–S, Li–O_2_, and Na-ion batteries.

## Graphical abstract

**Figure:**
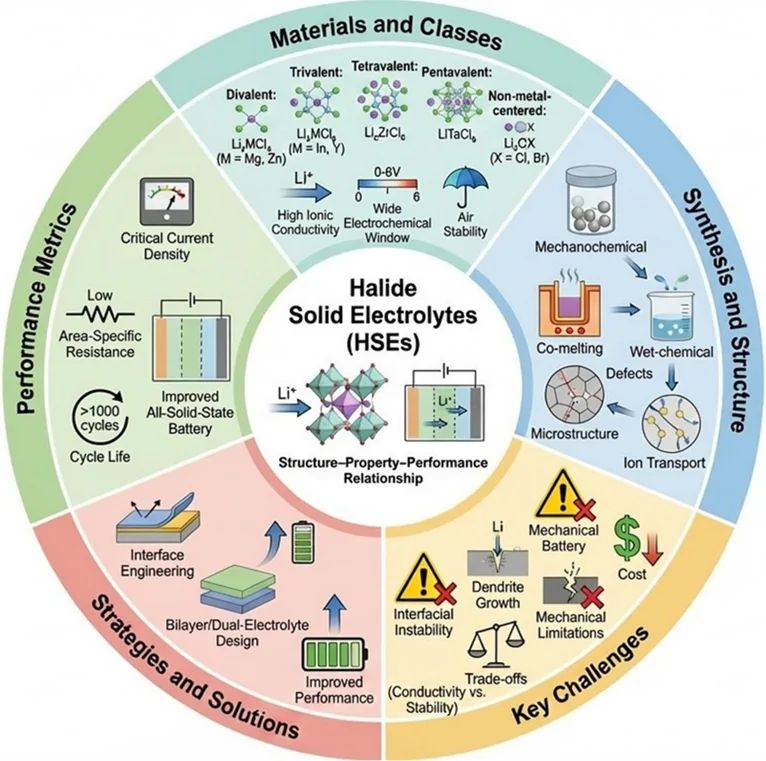

## Introduction

The increasing global energy demand for portable electronics, electric vehicles, and grid storage has demanded research into lithium-based/post-lithium batteries with higher-energy density, enhanced safety, and long-term reliability [1, 2]. Conventional lithium-ion batteries (LIBs) utilizing liquid electrolytes have exhibited practical energy densities of ~ 300 Wh kg^−1^. However, the inherent safety and stability problems of organic solvents, which are flammable, leak-prone, and have limited electrochemical stability windows, fundamentally restrict their performance. These electrolytes can experience thermal runaway under abusive conditions, producing heat and releasing dangerous gases, which raises serious safety concerns [3, 4]. All-solid-state lithium batteries (ASSLBs) based on solid-state electrolytes (SSEs) have emerged as a promising alternative to meet the ambitious targets of next-generation energy storage systems, which include energy densities of 400–500 Wh kg^−1^ and volumetric densities exceeding 650 Wh L^−1^.

The main change made to battery design is the substitution of SSEs for liquid electrolytes. Because SEs are naturally non-flammable, they can operate safely even at high voltages. They can provide wider electrochemical stability windows, often surpassing 5.0 V vs. Li/Li⁺, allowing the use of high-voltage cathodes such as LiNi_0.8_Co_0.1_Mn_0.1_O_2_ (NCM811) and LiCoO_2_ (LCO) without extensive cathode coating or buffer layers [5, 6]. Additionally, the high mechanical modulus of SEs controls lithium dendrite formation, which is vital for the long-term cycling stability of lithium metal anodes. These properties also support compact, bipolar cell designs, lowering the amount of inactive material content and potentially enabling volumetric energy densities in the range of 800–1000 Wh L^−1^ [7]. Collectively, these advantages highlight the potential of ASSLBs as a safer and more energy-dense replacement to conventional LIBs.

Up until now, SSE research has mostly concentrated on four material classes: oxides [8–12], sulfides [13–17], polymers [18–31], and halides [32, 33]. Garnet-type Li_7_La_3_Zr_2_O_12_ (LLZO) is an example of an oxide-based electrolyte with exceptional resistance to lithium dendrite growth and high chemical and electrochemical stability. However, their brittleness and need for high-temperature sintering limit their interfacial contact with electrodes, posing challenges for scalable manufacturing [34–36]. Sulfide-based electrolytes, such as Li_10_GeP_2_S_12_ (LGPS) and argyrodite Li_6_PS_5_Cl, exhibit elevated ionic conductivities (> 10 mS cm^−1^) and favorable deformability; however, these are highly sensitive toward moisture and release of toxic H_2_S, complicating handling and large-scale production [37, 38]. In the case of polymer electrolytes, they have good mechanical flexibility and ease of processing; however, they possess low ionic conductivity at room temperature and often require operation above 60 °C to attain satisfactory performance [39–43].

Recently, HSEs have become a promising class that bridges the gap between oxide and sulfide SSEs. They typically used Li_3_MX_6_, where M is a rare-earth element or a trivalent metal and X is a halide. These SEs could be the group that fills the gap between oxide and sulfide SSEs. These materials have moderate-to-high ionic conductivities (usually between ~ 10^–4^ to 10^–3^ S cm^−1^ at room temperature), wide electrochemical stability windows (> 4 V vs. Li/Li⁺), and are more stable in air than sulfide-based electrolytes [44–46]. Because HSEs are highly stable against oxidation, they can be directly connected to high-voltage cathodes. Their lattice is relatively soft, which makes them easier to work with and improves contact with active electrode materials [47–49]. Also, their different structures, such as divalent, trivalent, tetravalent, pentavalent, and non-metal-centered frameworks, enable modulation of ionic transport pathways and electrochemical properties, facilitating the design of high-performance ASSLBs.

Even though HSEs offer many benefits, significant scientific and technological challenges still prevent this type of SE from being used in practical applications. One of the biggest problems is that the HSE/Li–metal interface is unstable. Because of this problem in the cell, the electrolyte can cause unwanted reactions with the electrode’s active materials, creating high-resistance interphases at the electrode–electrolyte interface. This makes interfacial resistance higher during cycling [50–52].

A critical trade-off exists between ionic conductivity and electrochemical stability, necessitating systematic material design strategies. Implementing defect engineering, including introducing vacancies or structural disorder, may compromise the material’s structural robustness [53]. The limited supply and high cost of rare-earth elements like In, Y, and Sc make it difficult to scale up operations [54]. Also, we do not fully understand the chemo-mechanical properties of HSEs, such as elastic modulus, deformability, and stack pressure requirements, even though these factors directly affect interfacial contact, dendrite suppression, and cycling stability [55]. A significant gap remains in establishing systematic correlations among synthesis processes, resulting microstructures, and device-level performance metrics such as critical current density (CCD), area-specific resistance (ASR), and cycle life.

Recently, HSEs have been increasingly interested in post-Li-ion chemistries alongside Li-ion chemistry, including all-solid-state sodium-ion (Na-ion), lithium–sulfur (Li–S), and lithium–oxygen (Li–O_2_) batteries. This is due to their wide working and electrochemical stability windows, moderate-high ionic conductivity, and modifiable interfacial properties. Because of these advantages, these materials can be used to stabilize reactive active electrode materials and enable high-energy–density ASSBs in future battery systems [56–62].

**Fig. 1:**
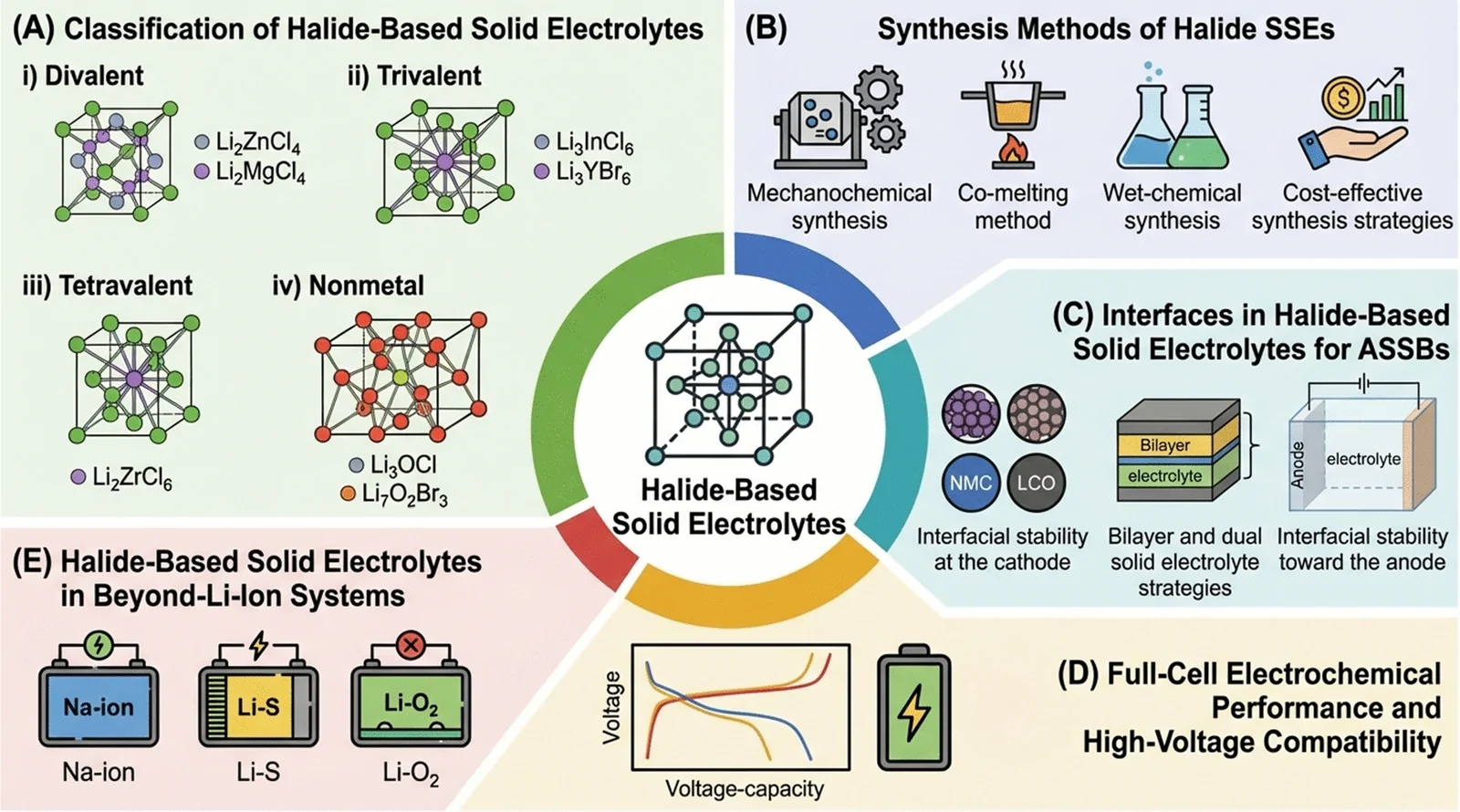
Overview of HSEs: classification, synthesis, interfaces, electrochemical performance, and applications in Li-ion and beyond-Li-ion batteries (Na-ion, Li–S, Li-O_2_)

In this context, this review aims to systematically address these unresolved challenges and knowledge gaps by providing a comprehensive and critical analysis of HSEs. We thoroughly classify HSEs based on the presence of a central metal, discuss synthesis strategies and their influence on defect chemistry and ionic conductivity, and emphasize interfacial modifications, including anode, cathode, and bilayer modifications, for stabilizing electrolyte–electrode interfaces. In addition, we established correlations between material synthesis, microstructure, and device-level performance and extended the discussion to emerging post-lithium battery systems. By integrating recent advances and identifying future research directions, this review provides a coherent framework for the rational design of halide SSEs toward safe, high energy density, and durable solid-state batteries. Figure 1 presents a graphical overview of the key aspects discussed in this work.

## Background and Classification of Halide-Based Solid Electrolytes

HSEs are a fast-emerging class of inorganic ionic conductors that are increasingly popular for ASSB applications. These materials are in general stable ternary or pseudo-ternary compounds with the general chemical formula Li_3_MX_6_, where *M* represents a central cation, and *X* denotes a halogen anion (F⁻, Cl^−^, Br^−^, or I^−^) [63]. High anion polarizability, tunable crystal chemistry, and relatively soft halide lattices combine to provide advantageous lithium-ion transport while maintaining broad electrochemical stability windows.

The literature confirms that HSEs exhibit moderate-to-high ionic conductivity at room temperature, typically ranging from 10^–4^ to > 10^–3^ S cm^−1^. Normally, conductivities > 1 mS m^−1^ (10^–3^ S cm^−1^) are achieved with ternary halide electrolytes, which are considered ideal for practical applications in ASSBs. Well-known examples are Li_3_InCl_6_ (~ 1–1.5 mS cm^−1^) and Li_3_YCl_6_ (~ 0.5–1 mS cm^−1^), which show high ionic conductivities comparable to other inorganic SEs.

**Fig. 2:**
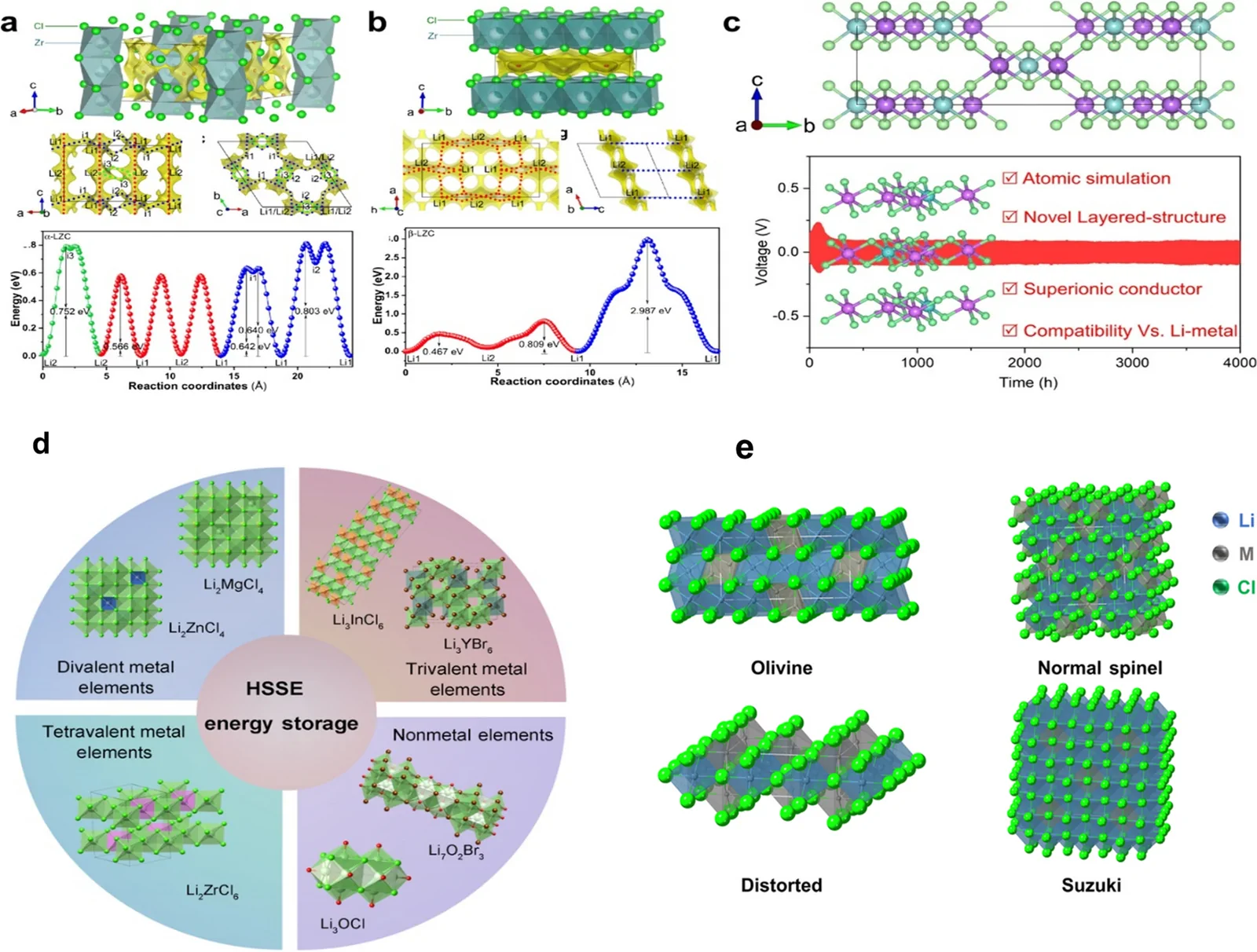
**a** Crystal structures of α-Li_2_ZrCl_6_. **b** β-Li_2_ZrCl_6_, overlaid with the Li-ion potential map. **c** Layered structural representation of Li_2_ZrCl_6_ and Li_2_HfCl_6_. Reproduced with permission from ref. [93]. Copyright 2026 American Chemical Society. **d** Classifications of existing halide SSEs and crystal structures of halide SSEs with **e** olivine-type, normal spinel, distorted spinel, and Suzuki-type frameworks. Reproduced with permission from Ref. [64]. Copyright 2024 Science Publications

In terms of ionic conductivity, mechanical compliance, and chemical stability, HSEs fall between oxide- and sulfide-based electrolytes. Structural chemistry indicates that the central cation M determines the coordination environment, lattice symmetry, lithium sublattice structure, and ultimately, how ions move. HSEs can be categorized into four primary groups according to the formal valence state of the central element: divalent, trivalent, tetravalent, and non-metal-centered halides [64] (Fig. 2d). This classification paradigm effectively facilitates the establishment of correlations among composition–structure–property links across diverse halide families.

### Divalent Central Metal Atom-Based HSEs

HSEs with divalent central metal atoms were among the first halide systems studied, and many studies were conducted on them in the 1970s and 1980s. These materials typically follow the chemical formula Li_2_MX_4_ (where X = Cl, Br and M = Mg, Ti, V, Cr, Mn, Fe, Co, Ni, Cd) and constitute the fundamental class of HSEs [63]. Li_2_MX_4_ compounds can be divided into four main structural types based on their crystallographic properties: olivine, normal spinel, distorted spinel, and Suzuki-type structures (Fig. 2e).

Most Li_2_MX_4_ halides crystallize in inverse spinel structures. These structures have a cubic close-packed (CCP) arrangement of halide anions and many ways for lithium to bond with other atoms. Li_2_MgCl_4_ is a good example because it has an inverse spinel structure in which Li⁺ ions occupy both tetrahedral and octahedral sites.

In particular, the three-dimensional halide framework is formed by one set of Li⁺ ions (Li_1_) occupying corner-sharing tetrahedral sites and the remaining Li⁺ ions (Li_2_) occupying edge-sharing (Mg_1_/Li_2_)Cl_6_ octahedra [65].

The structural polymorphism of divalent HEs is extremely sensitive to synthesis conditions and composition. For example, Li_2_ZnCl_4_ crystallizes at relatively low temperatures in a normal spinel structure. However, when heated to high temperatures, it undergoes a phase transition to an olivine-type structure. Likewise, Li_2_FeCl_4_ and Li_2_CoCl_4_ display distorted spinel structures, Li_6_FeCl_8_ and Li_6_CoCl_8_ crystallize in Suzuki-type frameworks [66–70]. Despite this wide structural variety, most stoichiometric Li_2_MX_4_ halides exhibit negligible lithium-ion conductivity at room temperature, thereby limiting their practical significance for solid-state battery applications.

A significant advancement in ionic transport was observed in nonstoichiometric spinel-type HEs with the general formula Li_2-2x_M_1+x_Cl_4_, such as Li_1.6_Fe_1.2_Cl_4_ and Li_1.52_Mn_1.24_Cl_4_ [71–73]. The ionic conductivity of these materials is noticeably higher than that of their stoichiometric counterparts. An increase in the number of mobile Li⁺ ions occupying tetrahedral sites and the introduction of lithium vacancies due to excess M^2+^ cations are responsible for the improvement. Together, these structural defects promote more efficient Li⁺ migration within the close-packed halide anion framework [63, 74]. Nevertheless, their room-temperature ionic conductivities typically remain limited to the order of ~ 10^–5^ S cm^−1^, which is still insufficient for high-performance ASSBs.

More recently, substantial progress has been made through the design of highly disordered, nonstoichiometric inverse spinel halide electrolytes. Notably, Nazar and co-workers reported Li_2_Sc_2/3_Cl_4_ and Li_2_In_1/3_Sc_1/3_Cl_4_, which exhibit room-temperature lithium-ion conductivities of 1.5 × 10^–3^ S cm⁻^1^ and 2.0 × 10^–3^ S cm⁻^1^, respectively, accompanied by low activation energies of 0.34 and 0.33 eV for Li⁺ diffusion [75, 76]. Although these materials retain an inverse spinel framework similar to that of Li_2_MgCl_4_, they are distinguished by a pronounced degree of lithium sublattice disorder. In these disordered inverse spinel systems, four distinct Li⁺ sites are identified: Li_1_, Li_2_, and Li_3_ located in face-sharing octahedral and tetrahedral environments, and Li_4_ occupying edge-sharing (Sc_1_/Li_4_)Cl_6_) octahedra. Three-dimensional Li⁺ transport is greatly improved by numerous energetically accessible lithium sites and interconnected diffusion pathways.

In comparison to both normal and stoichiometric inverse spinel structures, deficient inverse spinel-type HSE generally shows superior ionic conductivity. Defect engineering is emphasized as a crucial role in enhancing HSE performance since the presence of excess vacancies and highly disordered Li⁺ sublattices dominates the enhancement of Li⁺ transport within the closely packed halide frameworks.

Table 1 shows the crystal structures and ionic conductivities at room temperature of some common divalent-metal-based HSEs.

**Table 1:** Representative divalent-metal-based HSEs: their structure and ionic conductivity

Composition	Central Metal (M)	Crystal Structure	Li⁺ Sites/Features	Ionic Conductivity (S cm^−1^)	References
Li_1.6_Mg_1.2_Cl_4_	Mg^2+^	Inverse spinel	Li⁺ in tetrahedral and octahedral sites	3.4 × 10^–5^ at RT	[73]
Li_2_ZnCl_4_	Zn^2+^	Normal spinel	Ordered Li⁺ sublattice	2.95 × 10^–9^ at RT	[77]
Li_2_FeCl_4_	Fe^2+^	Distorted spinel	Limited Li⁺ mobility	1.9 × 10^–3^ at 200 °C	[78]
Li_2_CoCl_4_	Co^2+^	Distorted spinel	Limited Li⁺ mobility	10^–2^ at 300 °C	[71]
Li_6_FeCl_8_	Fe^2+^	Suzuki-type	Ordered framework	2.2 × 10^–4^ at 200 °C	[70]
Li_6_CoCl_8_	Co^2+^	Suzuki-type	Ordered framework	6.2 × 10^–5^ at 200 °C	[70]
Li_1.6_Fe_1.2_Cl_4_	Fe^2^⁺	Deficient inverse spinel	Li vacancies + tetrahedral Li⁺	1.3 × 10^–5^ at 20 °C	[72]
Li_1.52_Mn_1.24_Cl_4_	Mn^2^⁺	Deficient inverse spinel	High vacancy concentration	1.5 × 10^–5^ at 25 °C	[73]
Li_2_Sc_2/3_Cl_4_	Sc^3^⁺*	Disordered inverse spinel	Four Li⁺ sites, high disorder	1.5 × 10^–3^ at 30 °C	[75]
Li_2_In_1/3_Sc_1/3_Cl_4_	In^3^⁺/Sc^3^⁺*	Disordered inverse spinel	Vacancy-rich Li⁺ network	2.0 × 10^–3^ at 30 °C	[76]

^*^ Sc^3^⁺ and In^3^⁺ are trivalent compounds, but they are included here for comparison because they are based on divalent-type inverse spinel halides

Ionic conductivities in deficient and highly disordered inverse spinel halide electrolytes are 1–2 orders of magnitude higher than those in stoichiometric divalent halides. This shows how important vacancy engineering and Li⁺ sublattice disorder are for improving ion transport.

### Trivalent Central Metal Atom-Based HSEs

HSEs with a trivalent central metal atom have attracted significant attention because they are highly stable electrochemically and exhibit higher ionic conductivity at room temperature than other HSEs. These materials most often crystallize in orthorhombic (Pnma), hexagonal or trigonal (P-3m1), or monoclinic (C2/m) shapes.

In terms of structure, hexagonal and orthorhombic phases are derived from hexagonal close-packed (hcp) anion arrangements, while monoclinic halide electrolytes are generally associated with a ccp halide anion framework. Lithium-ion migration pathways and overall transport dimensionality are crucially governed by the underlying anion packing.

**Fig. 3:**
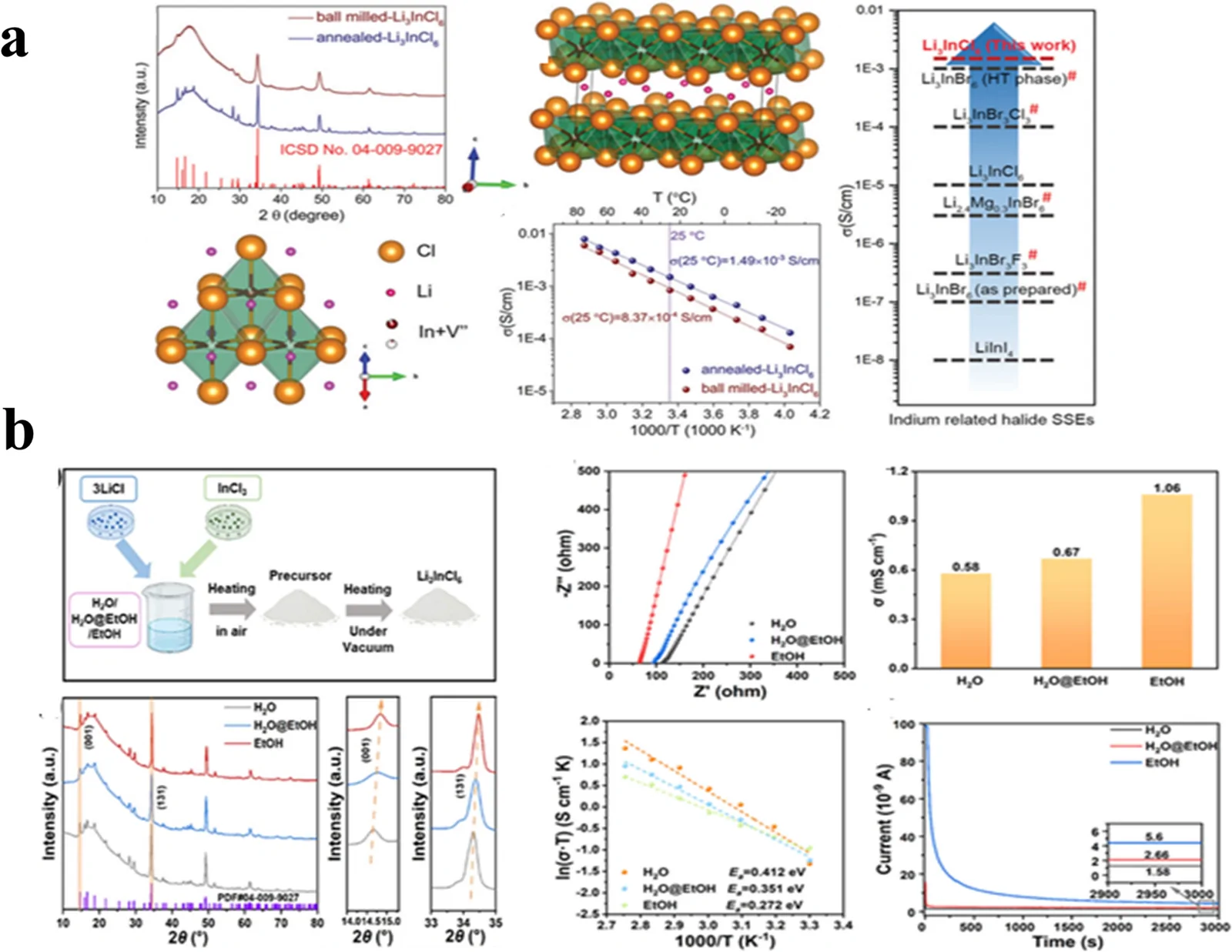
**a** XRD patterns of ball-milled and annealed Li_3_InCl_6_ compared with the standard Li_3_InCl_6_ pattern (ICSD No. 04–009-9027), crystal and layered structures of annealed Li_3_InCl_6_, schematic Li⁺ sites around InCl_6_^3−^ octahedra, Arrhenius plots, and a summary of reported indium-based HSEs. HT denotes high-temperature phase; bromide-based SSEs marked with “#” undergo structural transitions at 13–60 °C. Reproduced with permission from Ref. [79]. Copyright 2019 The Royal Society of Chemistry. **b** Solution-mediated synthesis of Li_3_InCl_6_ and comparison of phase structure (XRD), ionic and electronic conductivities, EIS spectra, and Arrhenius behavior for samples prepared using different solvents. Reproduced with permission from Ref. [80]. Copyright 2025, American Chemical Society

Among trivalent halide electrolytes, Li_3_InCl_6_ (LIC) has emerged as a prototypical system. Li et al. [79] synthesized LIC via a mechanochemical route using a stoichiometric mixture of LiCl and InCl_3_, followed by annealing at 260 °C for 5 h. X-ray diffraction (XRD) analysis revealed that the resulting material crystallized in a distorted monoclinic rock-salt-type structure with the C2/m space group. In this structure, Cl^−^ anions adopt a ccp arrangement, forming a three-dimensional framework that accommodates partially occupied Li⁺ sites and enables long-range lithium-ion transport. As a result, it is shown with a high Li^+^ conductivity of 1.49 × 10^–3^ S cm^−1^ at room temperature (Fig. 3a). More recently, Xiong et al. [80] reported the synthesis of LIC using a wet-chemical approach with different solvents, including water, ethanol, and mixed water–ethanol systems. The XRD patterns of all samples were well indexed to monoclinic Li_3_InCl_6_ (ICSD No. 04–009-9027, space group C2/m). Notably, the sample prepared via the ethanol-assisted route exhibited enlarged lattice parameters and a higher concentration of Li⁺ vacancies, indicative of enhanced structural disorder. This material largely crystallized along the (131) plane, which makes it easier for lithium ions to migrate in three directions. Because of this, it possessed the highest ionic conductivity at room temperature (1.06 mS cm^−1^) and the lowest activation energy for Li⁺ diffusion (0.272 eV) (Fig. 3b).

Li_x_ScCl_3+x_ (x = 2.5–4.0) [81] is another well-known family of trivalent halide electrolytes. When x is 2.5 or higher, the compositions crystallize in monoclinic Li_3_ScCl_6_ (C2/m, ICSD No. 04–009-8885). This has a ccp anion framework like LIC. These materials exhibit high ionic conductivity at room temperature, with Li_3_ScCl_6_ reaching 3.02 × 10^–3^ S cm^−1^. The improved transport is due to a perfect balance between mobile Li⁺ ions and lattice vacancies. This reduces the blocking effect of Sc^3+^ cations and makes it easier for Li⁺ ions to move in three dimensions, as in LIC.

In addition to chlorides, bromide- and iodide-based trivalent HEs have exhibited favorable ionic transport characteristics. Bromide compounds like Li_3_MBr_6_ (M = In, Sc, Y, Sm, Eu, Gd, Tb, Dy, Ho, Er, Tm, Yb, Lu) form a monoclinic C2/m structure with a ccp anion sublattice [63, 82]. In the same way, iodide-based conductors like Li_3_MI_6_ (M = Er, Sc, Y, La) have monoclinic structures, with Er-containing phases crystallizing in C2/c symmetry and other phases crystallizing in C2 space groups [83, 84]. The larger ionic radii of Br⁻ and I⁻ expand the lattice volume, reduce migration barriers, and promote three-dimensional lithium-ion conduction, often leading to improved room-temperature ionic conductivity compared with chloride analogs.

In addition to In^3+^ and Sc^3+^-based systems, other trivalent-cation HSEs, including Ti^3+^, Al^3+^, La^3+^, and V^3+^-based compounds, have been investigated, yielding a better understanding of structure–property interactions. Wang et al. [85] reported a Ti^3+^-based HSE, lithium titanium chloride (Li_3_TiCl_6_). The crystal structure is isostructural with LIC, crystallizing in the *C2/m* space group. They also demonstrated that Li_3_TiCl_6_ exhibits a room-temperature ionic conductivity of 1.04 × 10^–3^ S cm^−1^. In contrast, Xuan Li et al. [86] reported Al-substituted HSEs, Li_3_Al_*x*_Y_1−*x*_Cl_6_ (LAYC), and investigated the influence of Al substitution on the crystal structure and lithium-ion transport. Remarkably, Li_3_Al_0.7_Y_0.3_Cl_6_ exhibits a relatively high ionic conductivity of 1.05 × 10^–4^ S cm^−1^ at 25 °C, despite a high Al substitution ratio of 70%. The XRD patterns of LAYC match a monoclinic system with the C2/m space group, consistent with the previously reported Li_3_InCl_6_ standard pattern (ICSD no. 04–009-9027), and indicate a ccp arrangement of anions.

Similarly, M. Majumder [87] reported the synthesis of Li_3_LaCl_6_ using both wet-chemical and ball-milling methods. XRD patterns from both methods confirm the formation of a trigonal (or hexagonal) phase, consistent with previous reports on rare-earth HSEs and the reference ICDD card 01–012-0605 (LaCl_3_). The measured ionic conductivities were 7.14 × 10^–4^ and 6.84 × 10^–4^ S cm^−1^ for the wet-chemical and ball-milling methods, respectively.

Furthermore, Song et al. [88] reported a V^3+^-based HSE, Li_3_VCl_6_. XRD confirmed a monoclinic crystal structure with *C2/m* space group. They further measured the ionic conductivity at room temperature, which was found to be 7.5 × 10^–5^ S cm^−1^. In general, trivalent HSEs show that structural disorder, lithium vacancy engineering, and controlled anion packing are all important for fast ion transport. Monoclinic phases with more vacancies and disordered Li⁺ sublattices always have better ionic conductivity and isotropic migration pathways. This makes trivalent HEs one of the best types for high-performance ASSLBs. Table 2 shows some examples of trivalent-metal-based HSEs, along with their crystal structures and ionic conductivities.

**Table 2:** Representative trivalent-metal-based HSEs and their electrochemical properties

Composition	Central Metal (M^3^⁺)	Crystal Structure (Space Group)	Anion Packing	Ionic Conductivity (S cm^−1^), at RT	References
Li_3_InCl_6_	In^3^⁺	Monoclinic (C2/m)	ccp	1.49 × 10^–3^	[79]
Li_3_InCl_6_ (ethanol-assisted)	In^3^⁺	Monoclinic (C2/m)	ccp	1.06 × 10^–3^	[80]
Li_3_YCl_6_	Y^3^⁺	Hexagonal (P-3m1)	hcp	0.51 × 10^–3^	[89]
Li_3_YBr_6_	Y^3^⁺	Monoclinic (C2/m)	ccp	0.72 × 10^–3^	[89]
Li_3_ScCl_6_	Sc^3^⁺	Monoclinic (C2/m)	ccp	3 × 10^–3^	[81]
Li_3_ErCl_6_	Er^3^⁺	Trigonal (P-3m1)	–	0.3 × 10^–3^	[90]
Li_3_TiCl_6_	Ti^3+^	Monoclinic (C2/m)	ccp	1.04 × 10^–3^	[85]
Li_3_Al_0.7_Y_0.3_Cl_6_	Al^3+^/Ya^3+^	Monoclinic (C2/m)	ccp	1.05 × 10^–4^	[86]
Li_3_LaCl_6_	La^3+^	trigonal (or hexagonal) phase	–	7.14 × 10^–4^	[87]
Li_3_VCl_6_	V^3+^	Monoclinic (C2/m)	ccp	7.5 × 10^–5^	[88]

### Tetravalent Central Metal Atom-Based HSEs

HSEs containing tetravalent central metal cations, particularly Zr^4+^ and Hf^4+^, have recently attracted increasing attention owing to their favorable balance among ionic conductivity, chemical stability, and material cost [91, 92]. Wang et al*.* [92] reported a cost-effective and humidity-tolerant chloride solid electrolyte, Li_2_ZrCl_6_, which delivers a room-temperature ionic conductivity of 0.81 × 10^–3^ S cm^−1^. Remarkably, Li_2_ZrCl_6_ exhibits excellent moisture resistance, with both its crystal structure and ionic conductivity remaining largely unchanged after exposure to 5% relative humidity, a feature that significantly distinguishes it from many conventional halide electrolytes, which are highly moisture-sensitive.

To elucidate the structural evolution of Li_2_ZrCl_6_, neutron powder diffraction was employed to monitor phase transitions during thermal treatment. As shown in Fig. 2a, the *α*-Li_2_ZrCl_6_ phase is the most important phase in the material prepared at room temperature. A mixed α/β two-phase region forms between 277 and 350 °C as the temperature increases. As the temperature rises further, only one *β*-Li_2_ZrCl_6_ phase remains stable. Upon cooling to room temperature, the material retains the *β*-phase (Fig. 2b), which is important because it indicates that *α*-Li_2_ZrCl_6_ is a metastable phase formed by mechanochemical ball milling, while the *β*-phase is the thermodynamically stable structure.

In addition to experimental characterization, advanced computational modeling has yielded a deeper understanding of the structural and transport properties of tetravalent HSEs. They predicted a layered structural motif for both Li_2_ZrCl_6_ and Li_2_HfCl_6_ using a global neural network (SSW-NN) with a stochastic surface walking approach, as shown in Fig. 2c [93]. This layered structure facilitated lithium-ion mobility, which explains both their mobility and the interface’s stability. After optimizing its structure, Li_2_ZrCl_6_ showed an ionic conductivity of about 1.0 × 10^–3^ S cm^−1^, which further proved that it could be an efficient HSE.

Tetravalent halide electrolytes, such as Li_2_ZrCl_6_ and Li_2_HfCl_6_, have the potential to achieve simultaneous cost reduction, humidity tolerance, and competitive ionic conductivity through strategic compositional selection and structural engineering. These characteristics make tetravalent halide systems particularly attractive for scalable all-solid-state lithium battery applications and provide valuable design principles for future halide electrolyte development.

### Pentavalent Central Metal Atom-Based HSEs

Recently, HSEs containing pentavalent central metal cations, particularly Ta^5+^and Nb^5+^ have been of interest due to their availability of numerous binary lithium and sodium containing halides or chalcogenides that can form both amorphous compounds and also crystalline (oxy) halides with ionic conductivity > 10 mS cm^−1^ [94–96]. Chaupatnaik et al. [96] reported crystalline LiNbCl_6_ and LiTaCl_6_ HSEs. XRD results confirm that both compounds adopt a triclinic layered structure with a hexagonal AB-type close packing of chloride anions corresponding to a distortion of the $R\overline{3 }$ structure of Cr_2_Se_3_. They achieved an ionic conductivity of 1 × 10^–5^ S cm^−1^ for crystalline LiTaCl_6_ phase.

Similarly, the amorphous LiTaCl_6_ was reported by Ishiguro et al. [94]. XRD studies confirm the absence of diffraction peaks, indicating its glassy nature. Interestingly, the measured ionic conductivity was significantly higher, reaching 1.1 × 10^–2^ S cm^−1^, suggesting that such materials could serve as promising high-conductivity pentavalent HSE.

Further, Zhao et al. [97] reported a new pentavalent HSE, Li_3_Ta_3_O_4_Cl_10_. XRD studies confirm that a monoclinic unit cell with the space group of I2/a. The prepared material shows a high ionic conductivity, reaching up to 13.7 mS cm^−1^.

### Nonmetal Central Elements-Based HSEs

Nonmetal-centered HSEs are distinguished by excellent Li metal compatibility and strong reductive stability due to the absence of redox-active metals [98–100]. Early examples were limited by low ionic conductivity and narrow electrochemical windows. A major advance came with anti-perovskite Li_3_OX (X = Cl, Br), including Li_3_OCl and Li_3_OCl_0.5_Br_0.5_, exhibiting ionic conductivities of 0.85–1.94 mS cm^−1^ and Li⁺ migration barriers of 0.18–0.26 eV [101]. Thin-film fabrication further enabled stable cycling with Li metal, demonstrating self-stabilizing interphases [102–104].

Layered anti-perovskites such as Li_7_O_2_Br_3_ show ~ 10 × higher conductivity than Li_3_OBr [105], whereas lithium halide hydroxides (Li₂OHCl, Li_4_(OH)_3_Cl, Li₅(OH)₃Cl₂) remain < 0.01 mS cm⁻^1^ [100, 106, 107]. Cluster-anion-based designs (e.g., Li_3_OBH_4_, Li₃O(BF₄)_0.5_Cl_0.5_) are predicted to reach ~ 100 mS cm^−1^ but await experimental validation. Recently, vacancy-rich Li_9_N_2_Cl_3_ demonstrated 0.043 mS cm^−1^ conductivity along with air and Li metal stability [108].

Nonmetal-centered HSEs thus offer high reductive stability and promising Li metal compatibility, with ongoing work focused on enhancing ionic conductivity via defect and anion engineering.

### High-Entropy HSEs

High-entropy HSEs are typically composed of multiple metal cations and exhibit a configurational entropy of ΔS ≥ 1.5R (where R is the ideal gas constant). Metal cations with similar ionic radii but varying valence states are selected to facilitate the formation of solid solutions. The high-entropy effect can significantly increase structural disorder in the arrangement of Li⁺ ions and vacancies, leading to a substantial improvement in ionic conductivity as well as a reduction in activation energy [54, 109].

**Fig. 4:**
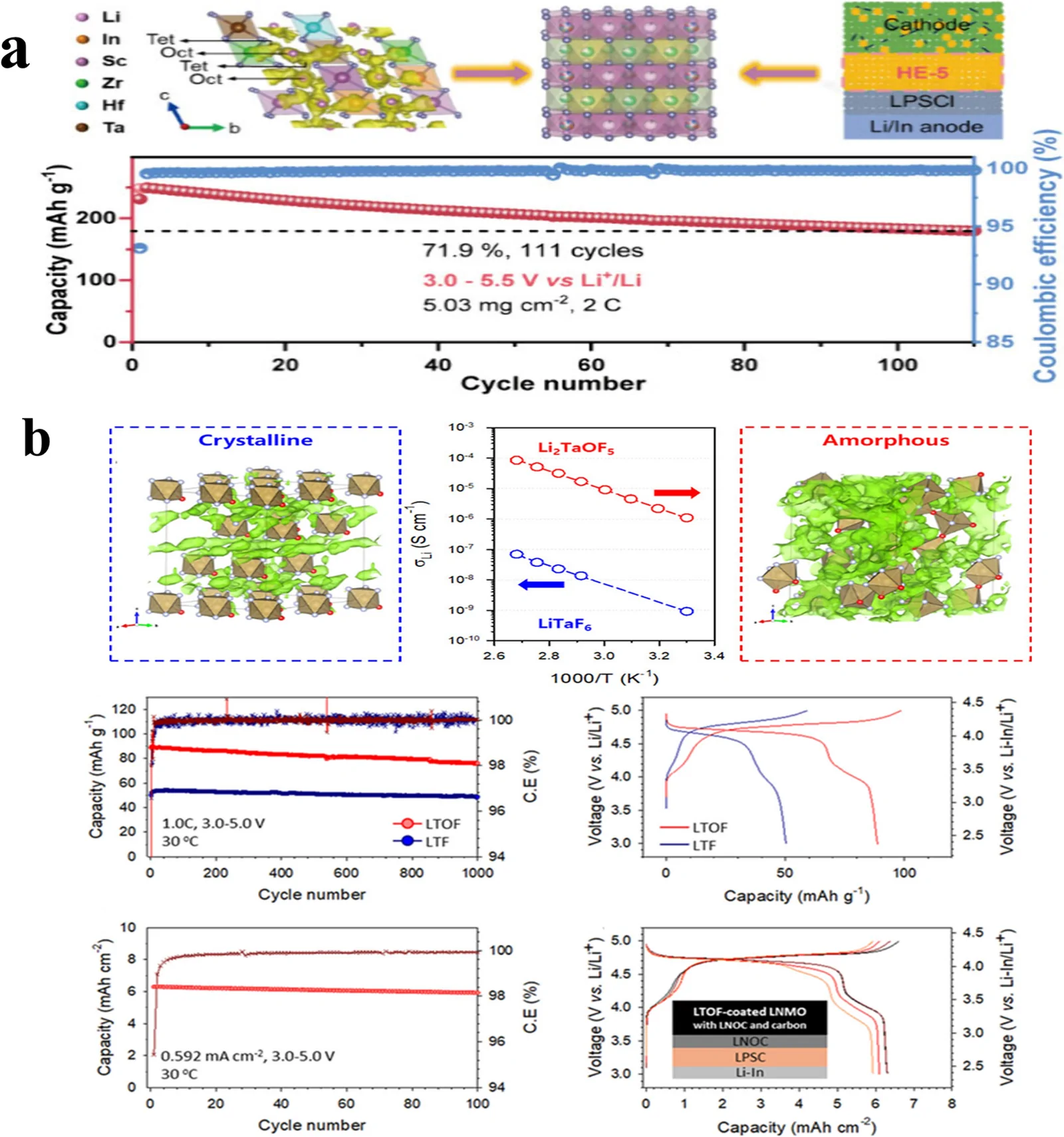
**a** Crystal structure of high-entropy solid electrolyte HE-5 (Li_2.2_In_0.2_Sc_0.2_Zr_0.2_Hf_0.2_Ta_0.2_Cl_6_) and its cycling performance with an NCM83 cathode and Li-In anode at 2 C and 5.5 V, including Coulombic efficiency. Reproduced with permission from Ref. [110]. Copyright 2021, American Chemical Society. **b** Crystal structures of LiTaF₆ and amorphous Li_2_TaOF_5_ (LTOF), their ionic conductivity, and electrochemical performance of LNMO cathodes using LTOF as a protective layer in all-solid-state cells at 30 °C, including cycling behavior and voltage profiles at various rates and areal capacities. Reproduced with permission from Ref. [113]. Copyright 2021, American Chemical Society

Recently, Ye et al. [110] reported a new high entropy HSE (HE-5, Li_2.2_In_0.2_Sc_0.2_Zr_0.2_Hf_0.2_Ta_0.2_Cl_6_) designed to improve ionic conductivity and high-voltage stability. The prepared HE-5 exhibits an ionic conductivity of 4.69 mS cm^−1^ at 30 °C and an activation energy of 0.300 eV. Furthermore, when integrated with a high-capacity NCM83 cathode and a Li-In anode, the material demonstrates 70% capacity retention over 1600 cycles at a 4 C rate, enabling stable operation at 5.0 V without significant degradation (Fig. 4a).

Similarly, Song et al. [111] introduced local lattice distortion by substituting In with multiple metal elements in Li_3_InCl_6_, resulting in a high-entropy Li_2.75_Y_0.16_Er_0.16_Yb_0.16_In_0.25_Zr_0.25_Cl_6,_ to address the high-voltage limitations of conventional HSEs. The induced lattice distortion modifies the distribution of Cl^−^ ions and promotes longer Li-Cl bonds, facilitating the favorable activation of Li^+^. The prepared HSE exhibits an ionic conductivity of 1.71 × 10^–3^ S cm^−1^, and ASSBs constructed with this high-entropy HSE shows 250% improvement over 500 cycles. In particular, the cell delivered a high discharge capacity of 185 mAh g^−1^ at a charge cutoff voltage of 4.6 V and a low current rate of 0.2 C. and furthermore,

Furthermore, Li et al. [112] employed a high-entropy approach to improve both ionic conductivity and high-voltage stability (> 4.8 V vs. Li^+^/Li) of HSEs. They designed a high-entropy SSE, (HE-SSE, Li_2.9_In_0.75_Zr_0.1_Sc_0.05_Er_0.05_Y_0.05_Cl_6_), which exhibits an ionic conductivity of 2.18 × 10^–3^ S cm^−1^. When coupled with an NMC532 cathode, the material demonstrates superior high-voltage and long-cycle stability, achieving 81.4% capacity retention over 250 cycles at 4.8 V (vs. Li^+^/Li), and up to 5000 cycles when battery operated at 4.6 V (vs. Li^+^/Li).

From these results, it can be concluded that high-entropy design effectively enhances ionic conductivity and high-voltage stability through increased structural disorder and lattice distortion. This strategy offers a promising pathway for the future development of high-energy–density durable ASSBs.

### Oxyhalide SEs

In recent years, oxyhalide-based SEs have emerged as promising SE systems. These materials often exist in an amorphous phase, in which metal polyhedra are connected over short distances via bridging oxygen atoms, thereby enhancing the ionic conductivity [54, 109].

Recently, Park et al. [113] reported a new class of amorphous oxyfluoride SE, Li_1+*x*_TaO_*x*_F_6–*x*_ (*x* = 0.0–1.0), which exhibits over three times higher Li^+^ conductivity than crystalline LiTaF_6_, reaching 1.08 × 10^–6^ S cm^−1^ at 30 °C (*x* = 1.0). XAS and Raman spectroscopy studies confirmed the formation of a corner-sharing chain framework of Ta(O/F)_6/7_ polyhedra. Furthermore, ASSBs assembled with Li_2_TaOF_5_ and 5 V-class LiNi_0.5_Mn_1.5_O_4_ cathodes demonstrated excellent cycling performance, retaining 85.8% capacity after 1000 cycles at 1.0 C and 30 °C. Even under high mass loading (49.3 mg cm^−2^) or low temperature (− 20 °C) conditions, the modified LNMO electrodes with Li_2_TaOF_5_ exhibited promising performance, achieving areal capacities > 5.9 mAh cm^–2^ with 94% retention (Fig. 4b).

Similarly, Xue et al. [114] developed a Li-*M*-X_5_ oxyhalide chemistry (Li_3*x*_TaO_3*x*_Cl_5–3*x*_, 0.8/3 ≤ *x* ≤ 1.4/3) to enhance the conductivity of SEs. The optimized oxyhalide SE achieves a high ionic conductivity of 9 mS cm^−1^ at 30 °C and 0.59 mS cm^−1^ at −35 °C. XRD, Raman spectroscopy, XPS, and XAS studies confirm that oxygen incorporation reduces Ta-O/Ta-Cl coordination numbers and induces distorted O/Cl-containing polyhedra, thereby facilitating Li^+^ ion migration. To further evaluate its practical applicability, ASSBs were assembled using a Li_1.2_TaO_1.2_Cl_3.8_ as the oxyhalide SE, single-crystalline LiNi_0.8_Co_0.1_Mn_0.1_O_2_ (NCM811) as the cathode, and a Li-In as the anode. The constructed battery showed outstanding cycling performance, delivered 100% capacity retention with a CE of 100%, after 3200 cycles at a voltage window of up to 4.2 V.

Additionally, Seung Kim et al. [115] introduced WO_2_Cl_2_ into Li_2_MCl_6_ (M = Y, Er and In), enabling the ordered introduction of oxygen into a diverse oxyhalide SEs, Li_2_M_1-x_W_x_Cl_6-2x_O_2x_ (x = 0.02, 0.04, 0.06, 0.08, 0.10). This method was employed to boost ionic conductivity and improve structural disorder. With the help of structural, spatial, and vibrational analyses, including synchrotron X-ray techniques, depth-resolved spectroscopy, and Raman measurements, confirmed that the incorporation of oxygen via [WO_2_Cl_4_]^2−^ polyhedral units, thereby affirming the structural integrity of the modified halides.

They additionally investigated air stability and conductivity measurements for all compositions. Zr-based samples demonstrated superior performance, exhibiting conductivities ranging from 1.15 × 10^–7^ to 5.56 × 10^–7^ cm^2^ s^−1^, as well as improved air stability. ASSBs constructed utilizing LiNi_0.6_Co_0.2_Mn_0.2_O_2_ (NCM622) cathodes exhibited that the Zr-based system presented the superior electrochemical performance.

### Trade-off Between Ionic Conductivity and Electrochemical Stability

A crucial problem in advancing HSE development is the intrinsic trade-off between ionic conductivity and electrochemical stability. Strategies that improve ionic conductivity, including increasing lattice polarizability, inducing structural disorder, and generating lithium vacancies, facilitate fast Li⁺ transport but may also adversely affect electrochemical stability, particularly under high-voltage (oxidative) or lithium metal (reductive) conditions. For instance, highly conductive trivalent and high-entropy halides typically exhibit elevated susceptibility to oxidative degradation at the cathode or to interfacial instability with a lithium metal anode. Conversely, systems characterized by robust bonding frameworks or non-metal-centered chemistries typically exhibit enhanced electrochemical stability windows but suffer relatively lower ionic conductivity. This trade-off is further evident at electrode–electrolyte interfaces, where highly conductive yet less stable electrolytes tend to form resistive interphases, thereby limiting long-term cycling performance. Therefore, achieving an optimal balance between ionic transport and electrochemical robustness remains a central challenge in the design of HSEs for practical ASSB applications.

## Synthesis Methods of HSEs 

The practical development of HSEs relies on synthesis routes that are not only scalable and cost-effective but also capable of bringing the desired microstructure and enhanced electrochemical properties. Currently, HSEs are primarily prepared via mechanochemical, co-melting, and wet-chemical approaches. Each method produces distinct structural characteristics in terms of crystallinity, grain size, and defect distributions.

These features critically influence Li-ion transport and interfacial characteristics, which directly govern device-level performance metrics such as area-specific resistance (ASR), critical current density (CCD), and cycle life. Therefore, establishing explicit correlations between synthesis methods, resulting microstructures, and practical electrochemical performance is essential for advancing HSE-based ASSBs.

### Mechanochemical Synthesis

Mechanochemical synthesis, most commonly implemented through high-energy ball milling, represents the most extensively employed and versatile route for preparing HSEs due to its unique advantages, such as simplicity, scalability, and ability to generate non-equilibrium structures [116]. In this technique, stoichiometric mixtures of lithium halides and metal halide precursors are placed in a sealed milling chamber and agitated vigorously. Repeated high-energy collisions cause particles to break apart, cold-weld, and mix at the atomic level. This speeds up solid-state reactions even when the temperature is not high [117]. The method is very effective for making trivalent and tetravalent HSEs because it allows regulating the material composition and defect chemistry in the structure, while losing a small amount of lithium precursor.

The development of amorphous or low-crystallinity metastable phases with high defect concentrations and smaller particle sizes is a key feature of mechanochemically produced HSEs. The resulting structural disorder is highly beneficial for ionic transport because it increases lithium vacancies, disrupts the long-range ordering of immobile cations, and facilitates the formation of isotropic, three-dimensional Li^+^ conduction pathways within the close-packed halide anion framework. Consequently, relatively high ionic conductivities on the order of ~ 10^–3^ S cm^−1^ are commonly achieved.

This enhanced ionic transport, combined with increased defect density and improved particle contact, directly contributes to reduced interfacial resistance (lower ASR), improved tolerance to higher current densities (enhanced CCD), and prolonged cycle life in ASSBs.

Recent research conducted by Samanta et al. [117] revealed that a one-step mechanochemical milling method can be employed to make a wide range of phase-pure HSEs, such as Li_3_YCl_6_, Li_3_InCl_6_, Li_3_ScCl_6_, Li_3_ErCl_6_, and Li_2_ZrCl_6_. Their findings highlight that mechanochemical processing facilitates the rapid synthesis of trivalent and tetravalent HSEs defined by excellent compositional uniformity, adaptable lithium vacancy ratios, and ionic conductivities on the order of ~ 10^–3^ S cm^−1^ in ambient environments. In this work, a series of bilayer cells were constructed to evaluate interfacial compatibility and electrochemical stability. Among them, the LiCoO_2_:Li_3_YCl_6_|Li_3_YCl_6_ | Li_6_PS_5_Cl | Li-In bilayer cell exhibited the best electrochemical performance, retaining 75% capacity after 100 cycles, demonstrating that mechanochemically induced structural homogeneity and defect engineering can mitigate interfacial degradation and improve cycle life.

Recently, Yang et al. [118] reported a mechanochemically synthesized, defect-containing series of HSEs, Li_3–3*x*_Y_1+*x*_Cl_6_ (0 < *x* < 0.17). For comparison, the same series of materials was also prepared by a conventional solid-state method. Among these compositions, Li_2.61_Y_1.13_Cl_6_ synthesized by the mechanochemical method exhibited the highest conductivity of 0.47 mS cm^−1^. Furthermore, ASSBs were constructed using LiNi_0.8_Mn_0.1_Co_0.1_O_2_ (NMC811) as the cathode. The resulting cells demonstrated excellent cycling stability, with ∼90% capacity retention after 1000 cycles, highlighting the beneficial impact of mechanochemical processing on long-term device performance.

To further evaluate the influence of the mechanochemical method on CCD, Ganesan et al. [119] explored the effect F substitution on conductivity and CCD of Li_2_ZrCl_6._ The Li_2_ZrCl_6-x_F_x_ (*X* = 0, 0.25, 0.50, 0.70, 1.00, and 1.20) series of HSEs via a mechanochemical method. The room-temperature ionic conductivities were measured as for Li_2_ZrCl_6_ (2.8 × 10^−4^ S cm^−1^) and 2.6 × 10^−4^, 2.1 × 10^−4^, 1.6 × 10^−4^, 1.0 × 10^−4^, and 8.4 × 10^–5^ S cm^−1^ for *x* = 0, 0.25, 0.50, 0.70, 1.00, and 1.20, respectively. The steady decrease in ionic conductivity with increasing F substitution was observed, due to stronger Li–F bonds, which could hinder the Li^+^ diffusion. Furthermore, CCD measurements were conducted over a current range of 0.1 to 0.7 mA cm^−2^. Li_2_ZrCl_6_ showed an irregular stripping/plating voltage profile at a current density of 0.6 mA cm^−2^ and short-circuited at 0.7 mA cm^−2^. In contrast, Li_2_ZrCl_5.5_F_0.5_ showed a relatively stable stripping/plating profile at all applied current densities, without any indication of dendrite formation. These results indicate that compositional tuning via mechanochemical synthesis can enhance dendrite resistance and improve CCD, even when ionic conductivity is moderately reduced.

Additionally, to understand ASR performance in the mechanochemical synthesis method, Umeshbabu et al. [120] reported a series of Li_2_ZrF_6–*x*_Cl_*x*_ (*x* = 0, 0.25, 0.5, 0.75, 1.0, 1.25, 1.5, and 2.0) HSEs by the mechanochemical method. This work examined the effect of Cl substitution on the conductivity and ASR of Li_2_ZrF_6._ The ionic conductivity studies confirm that Li^+^ conductivity increases with increasing Cl content in Li_2_ZrF_6–*x*_Cl_*x*_. Compared to *x* = 0, Li^+^ conductivity for the composition with *x* = 1 improved by ∼5 orders of magnitude. Particularly, Li^+^ conductivities for Li_2_ZrF_5_Cl_1_ at 25 and 100 °C are 5.5 × 10^−7^ and 2.1 × 10^−5^ S cm^−1^, respectively. Further, conducted interface studies with Li/Li_2_ZrF_5_Cl_1_/Li cell, at RT cell shows large ASR of 82.5 kΩ cm^2^ and at the cell at 100 °C, a lower interfacial ASR of 6.1 kΩ cm^2^ is obtained, which is almost 13.4 times smaller than RT interfacial ASR. This significant reduction in ASR highlights the strong dependence of interfacial resistance on both composition and synthesis-induced microstructure.

Despite these benefits, excessive mechanical activation during milling might cause over-amorphization, crystal structure collapse, or contamination from the milling media. This could potentially affect the long-term electrochemical stability and reproducibility of the SSEs. Therefore, to find the best balance between defect generation and stable structure, it is important to carefully optimize the milling parameters, milling duration, rotational speed, milling atmosphere, and ball-to-powder ratio [116]. Well-controlled mechanochemical synthesis is a highly effective and scalable way to produce high-performance HSEs for advanced ASSBs.

### Co-Melting Method for HSEs 

**Fig. 5:**
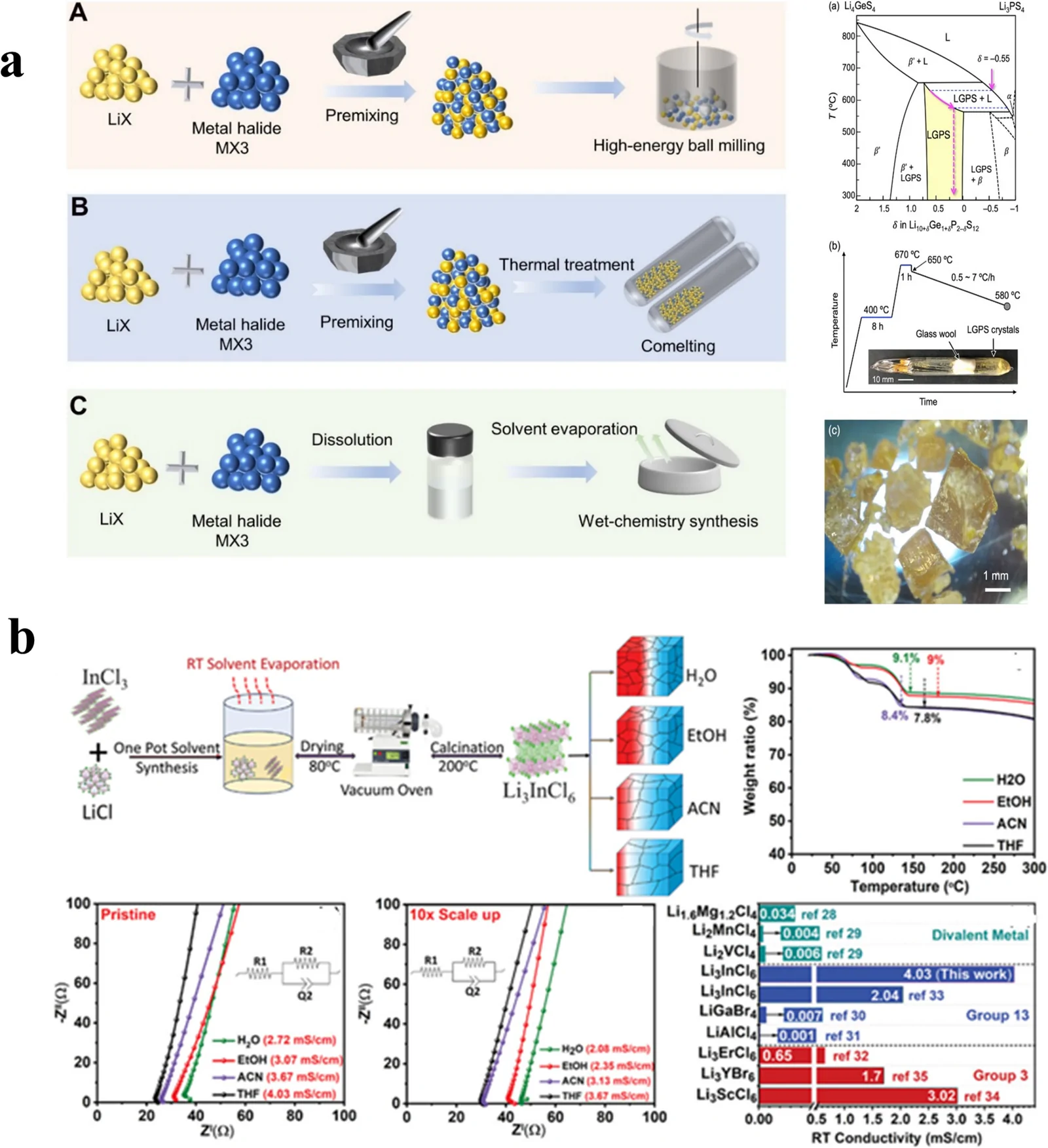
**a** Synthesis methods of HSEs: (A) high-energy ball milling, (B) co-melting, and (C) wet-chemical synthesis (left panel). Reproduced with permission from Ref. [122]. Copyright © 2022, Science. Right panel shows the Li_4_GeS_4_-Li_3_PS_4_ pseudo-binary phase diagram, where magenta arrows indicate the applied cooling pathway starting from the liquid phase (δ = − 0.55), together with a photograph of typical millimeter-sized LGPS crystals. Reproduced with permission from Ref. [124]. Copyright 2022, American Chemical Society. **b** Schematic illustration of the wet-chemical synthesis of Li_3_InCl_6_ SSEs, corresponding thermogravimetric analysis (TGA) of intermediates after drying at 80 °C, Nyquist impedance plots of Li_3_InCl_6_ synthesized via the wet-chemical route and the 10 × scaled-up THF-derived samples, and comparison of room-temperature (22 °C) ionic conductivities with reported Li-ion-conducting halide SSEs measured on cold-pressed pellets. Reproduced with permission from Ref. [125]. Copyright 2022, Wiley

The co-melting synthesis route represents a thermodynamically controlled and structurally robust approach for preparing HSEs, particularly for trivalent halide systems such as Li_3_MCl_6_, and LiMCl_6_ (M = Ta/Nb) [55, 96, 121]. In this method, stoichiometric mixtures of lithium halides (LiCl, LiBr, or LiI) and corresponding metal halides (MCl_3_, MBr_3_, or MI_3_) are thoroughly mixed, hermetically sealed in evacuated or inert-gas-filled ampoules, and subsequently heated above the eutectic or melting temperatures defined by their respective phase diagrams (Fig. 5a) [122]. Complete melting enables atomic-scale homogenization of the constituent species and promotes the formation of equilibrium crystalline phases with long-range structural order.

One of the principal advantages of the co-melting approach is its ability to produce highly crystalline halide SSEs with large grain sizes and a low density of grain boundaries, thereby minimizing grain-boundary resistance and allowing intrinsic lithium-ion transport to dominate the overall ionic conductivity [116]. This reduction in grain-boundary resistance directly contributes to lower ASR and improved rate capability in ASSBs, as ion transport is dominated by intrinsic bulk conduction rather than interfacial limitations. This is an important feature for HSEs because grain-boundary effects can mask the intrinsic transport properties of the close-packed anion framework. Also, fully controlling the heating profile, including the melting temperature, dwell time, and cooling rate, is an effective way to alter cation ordering and the distribution of lithium vacancies. Both factors have a significant effect on the rate at which Li⁺ moves through halide lattices [76]. This decrease in grain-boundary resistance directly reduces ASR and improves the rate capability of ASSBs because intrinsic bulk conduction, rather than interfacial limitations, controls ion transport.

Co-melting synthesis has been demonstrated to stabilize either monoclinic (C2/m) or hexagonal (P-3m1) crystal structures in Li_3_MCl_6_ systems, characterized by well-defined cubic close-packed (ccp) or hexagonal close-packed (hcp) anion sublattices, depending upon the nature of the central metal cation [79]. Similarly, Li et al. [121] also prepared Li_3_Ho_1-x_In_x_Cl_6_ HSE using a co-melting synthesis method, which enabled the development of well-defined crystalline phases. The materials showed a clear transition from hcp-T to hcp-O, confirming theoretical predictions. The relative amounts of Li⁺ and M^3^⁺ cations decide this modification. These cations influence the amount of vacancies required for fitting stacked cations into the halide anion framework.

Compared with hcp-T Li_3_YCl_6_, the hcp-O phases displayed significantly higher room-temperature ionic conductivities (5.7- 6.9 × 10^–4^ vs. 7.1 × 10^–5^ S cm^−1^), attributed to enhanced Li⁺ diffusion along the *z*-direction. A similar increase in conductivity was observed in Li_2.727_M_1.091_Cl_6_ (M = Dy, Ho, Y, Er, Tm), indicating that co-melting-induced structural engineering can be used to improve ion transport in HSEs in general.

Recently, Tao et al. [123] reported spinel-type HSEs with compositions Li_2-2*x*_Mn_1+*x*_Cl_4_ (*x* = 0–0.3), prepared via the co-melting method. The authors proved that by tuning the Li/Mn ratio, enhanced Li vacancies, Li + diffusion, and spinel lattice reconstruction were achieved. The optimized Li_1.7_Mn_1.15_Cl_4_ (*x* = 0.15) exhibited a RT ionic conductivity of 0.12 mS cm^−1^. Furthermore, interfacial compatibility with Li metal was systematically studied. The Li | Li_1.7_Mn_1.15_Cl_4_ | Li symmetric cell cycled over 250 h at CCD of 0.05 mA cm^−2^ without voltage fluctuation. In addition, a constructed ASSB cell employing NCM811-Li_1.7_Mn_1.15_Cl_4_ as the composite cathode, Li-In alloy as the anode, and Li_1.7_Mn_1.15_Cl_4_ as the SE. The cell achieved an initial CE of 90.51% at 0.05 C/50 °C and maintained a stable charge/discharge cycle over 60 cycles. The stable cycling behavior and CCD performance demonstrate that co-melting-derived crystalline structures can provide improved interfacial stability and sustained electrochemical performance.

A representative example from sulfide-based HSEs highlights the key concepts of co-melting synthesis. Iwasaki et al*.* reported the synthesis of Li_10_GeP_2_S_12_ (LGPS) employing a pseudo-binary phase diagram of Li_4_GeS_4_-Li_3_PS_4_. In this process, stoichiometric powders of Li_2_S, Ge, P, and S were mixed together in an argon-filled glovebox, pressed into pellets, sealed in quartz ampoules, and subjected to a carefully executed thermal procedure that included complete melting and regulated cooling [124] (Fig. 5a).

Even with these advantages, several problems remain with the co-melting synthesis of HSEs. To melt lithium, you need to heat it to a high temperature, but this can cause it to evaporate, allow halogens to leak out, or break the sealed ampoule. To prevent these issues, it is necessary to have strict control over the external environment, sealing conditions, and thermal ramping rates [116]. Also, while fast quenching may be needed to retain metastable high-conductivity phases, slow cooling could lead to excessive cation ordering, slowing Li⁺ transport. These things show how important it is to use phase-diagram-guided synthesis and careful temperature optimization when making HSEs with the co-melting approach, which involves carefully planned thermal processes that include complete melting and controlled cooling.

In general, the co-melting method is an excellent method to investigate the link between structure and transport in HSEs and to prepare benchmark materials with a highly crystalline structure. However, its relatively high-energy consumption, scalability limitations, and batch-limited throughput make it necessary to continue searching for alternatives to produce HSEs on a large scale, particularly mechanochemical and wet-chemical methods [63, 76].

### Wet-Chemical Synthesis

Wet-chemical synthesis has become a scalable and potentially cost-effective method for preparing HSEs, with benefits that are hard to achieve with traditional solid-state or co-melt-based synthesis methods. Solution-mediated processes allow for exact control over the size of the crystallites, the purity of the phase, the morphology, and the distribution of defects. All of these things have a direct effect on the density of the grain boundaries, the local macrostrain, and the way lithium ions move in HSEs [63, 122]. Such control over microstructure and grain-boundary characteristics plays a critical role in minimizing ASR and enhancing long-term cycling stability. Wet-chemical methods offer a flexible framework for adjusting local structural disorder and enhancing lithium-ion diffusion pathways within halide lattices by meticulously controlling solvent coordination strength, precursor solubility, and reaction kinetics.

Bonsu et al. [125] recently reported a wet-chemical synthesis strategy for highly conductive Li_3_InCl_6_ HSE. They reached ionic conductivities > 2 mS cm^−1^ at room temperature and a record value of ~ 4 mS cm^−1^ at 22 °C. This is one of the highest conductivities for halide-type SEs that have been experimentally recorded so far. To elucidate the influence of solvents on phase formation and microstructural evolution, they carried out a systematic investigation employing both protic (ethanol and H_2_O) and aprotic (acetonitrile and tetrahydrofuran, THF) solvents. The results demonstrated that aprotic solvent-mediated synthesis successfully minimizes grain-boundary resistance and mitigates microstrain inside the Li_3_InCl_6_ lattice by yielding significantly larger crystallite sizes and more agglomerated particle morphologies.

As a result, Li_3_InCl_6_ synthesized with THF demonstrated significantly improved bulk lithium-ion conduction, with intrinsic lattice diffusion predominating over intergranular pathways in ionic conduction. The Li_3_InCl_6_ prepared by using THF solvent only worked better electrochemically, but it also showed good air stability. When the material was exposed to humid conditions, it showed minimal structural changes and minimal changes in conductivity. These results show how important solvent chemistry is for controlling microstructural evolution and defect engineering during wet-chemical synthesis. They also show that this method is an excellent strategy to improve ionic conductivity, microstructural integrity, and moisture tolerance in HSEs all at once [125] (Fig. 5b). Furthermore, the authors investigated interfacial stability in both protic and aprotic solvents. In the case of aprotic solvents, the ACN (60 Ω) and THF (50 Ω) cells show significantly lower interface resistance than protic solvents, H_2_O (90 Ω) and EtOH (100 Ω), respectively. They also constructed ASSB with LiNi_0.6_Mn_0.2_Co_0.2_O_2_ cathode with a THF-mediated route SE. The cell delivered a capacity retention of 85% after 1000 cycles at 60 °C with a high 99.75% Coulombic efficiency. These results demonstrated that the THF-mediated synthesis route substantially enhances interfacial stability and long-cycling performance, thereby directly reducing ASR, improving cycle life, and enabling stable high-rate operation.

Recently, Yan et al. [126] reported a bihalogen doped (Br and F) co-doped Li_3_YCl_6_ system prepared via a wet-chemical method, with a final composition of Li_3_YBr_2_Cl_3.7_F_0.3_. The doping of Br and F was presented to improve both ionic conductivity and interfacial stability. The co-doped composition exhibited an ionic conductivity of 2.38 × 10^–3^ S cm^−1^, compared to 3.04 × 10^–3^ S cm^−1^ for pristine Li_3_YCl_6._ Interfacial stability studies further confirmed that the co-doped HSE offered improved Li plating/stripping activity, continued stable cycling over 1600 h at a current 0.1 mA cm^−2^. Authors further constructed ASSBs using an NCM811 cathode to assess device-level performance. Cell with pristine SE delivered an initial discharge capacity of 140 mAh g^−1^ with a CE of 71.9%, whereas the co-doped SE achieved a higher initial discharge capacity of 185 mAh g^−1^ and an improved CE of 75.7% at 0.1 C. Additionally, the co-doped SE showed enhanced cycling stability over 100 cycles, retaining a capacity of 70 mAh g^−1^ at 0.3 C.

In addition, Li_3_InCl_6_ has been widely recognized as a well-known crystalline, trivalent-metal-centered HSE, and numerous important studies have further highlighted the correlation between synthesis methods and its conductivity. Li et al. [127] reported a significant breakthrough as they revealed a water-mediated synthesis route with an ionic conductivity of 2.04 mS cm^−1^ at room temperature. This established water-based processing as a scalable and cost-effective method for HSEs. Furthermore, various solvent systems, which include ethanol, acetonitrile, ammonia and water–ethanol mixed solvents, have been studied to control over crystallization kinetics, defect chemistry, and lithium vacancy formation, typically yielding conductivities between ~ 0.7 and 1.25 mS cm^−1^, depending on synthesis conditions [80, 128–132]. The results collectively demonstrate that solvent chemistry significantly influences the ionic conductivity of Li_3_InCl_6_, suggesting an in-depth assessment of different solvent systems to account for recent improvements in the wet-chemical synthesis of HSEs.

### Cost-Effective Synthesis Strategies for Industrial Applications

The possibility of synthesis methods that integrate superior electrochemical performance with minimal energy consumption, greater material yield, and industrial scalability is essential for the practical application of HSEs in ASSBs. Because of this, low-temperature mechanochemical and solution-based synthesis methods have become highly regarded for cost-effective production. This is because this method has lower processing temperatures, synthesis times, and lithium or halogen loss by considerably in comparison to traditional high-temperature solid-state or melt-based methods. For developing batteries on a large scale, these benefits mean lower production costs and better control over the composition.

Direct solid-state reactions that are not in equilibrium are feasible through mechanochemical synthesis. This eliminates the need for lengthy calcination processes and significantly reduces the loss of lithium and halide species. This method optimizes the utilization of precursors and generates structures with several defects that are conducive to rapid Li⁺ transport. Mechanochemical processing is inherently compatible with scalable and continuous industrial platforms, including novel methods like twin-screw extrusion and large-volume ball milling. This is an excellent method for producing HSE granules in large quantities with consistent properties [133–135].

Solution-mediated synthesis is an additional cost-effective method for the preparation of materials, as it enables precursors to combine and precipitate at low temperatures. This method gives you precise control over the size distribution of the particles, the crystallinity, and the defect chemistry. This lowers grain-boundary resistance and increases bulk ionic conductivity. Wet-chemical processing can easily be added to existing chemical engineering processes, like continuous-flow reactors, spray drying, and solvent recovery and recycling systems. This lowers costs and environmental impact even more while making processes more sustainable [136, 137].

Alternatively, co-melting and sealed ampoule synthesis methods have inherent drawbacks such as high-energy consumption, batch-limited throughput, and strict atmosphere control requirements, despite being very successful in creating phase-pure and structurally well-defined benchmark materials. Their industrial scalability and economic viability are severely limited by these factors. Thus, a practical and financially advantageous route to the large-scale production of HSEs for next-generation commercial ASSBs technologies is the combination of mechanochemical and wet-chemical synthesis techniques.

In general, these examples show that synthesis strategies are not merely steps in the preparation process; they also significantly affect the performance of HSE-based ASSBs at the device level. Mechanochemical methods enable defect engineering and improved ionic transport, thereby increasing CCD and cycle life. Co-melting methods produce materials with many crystals, which have lower grain-boundary resistance and lower ASR. Wet-chemical methods allow you to control microstructure and interfacial properties with high precision, leading to better cycling stability and lower interfacial resistance. So, it is important to choose and optimize synthesis routes logically to produce high-performance ASSBs with balanced ionic conductivity, stable interfaces, and long-term durability. Table 3 lists the main methods for making HSEs and shows how each affects microstructure, ionic conductivity, and device-level performance metrics such as ASR and cycling stability.

**Table 3:** Comparison of bilayer electrolyte strategies in halide-based ASSBs

Synthesis Method	Key Features	Advantages	Representative HSEs	Ionic Conductivity (S cm^−1^)	References
Mechanochemical (High-energy ball milling)	Stoichiometric halides milled at high energy; formation of amorphous or low-crystallinity, defect-rich phases with high Li⁺ vacancy concentration	Scalable, rapid, precise composition control; enhanced ionic transport due to defect engineering; improved interfacial contact leading to reduced area-specific resistance (ASR)	Li_3_YCl_6_, Li_3_InCl_6_, Li_3_ScCl_6_, Li_3_ErCl_6_, Li_2_ZrCl_6_	1–2 × 10^–3^ at RT	[79, 117]
Co-melting/Melt synthesis	Halides melted in sealed ampoules above eutectic/melting temperature; slow cooling to form highly crystalline, equilibrium phases with long-range order	Produces large-grain materials with low grain-boundary density; enhanced bulk ionic conductivity and reduced grain-boundary resistance, contributing to lower ASR and improved rate capability	Li_3_InCl_6_, Li_3_Ho_1-x_In_x_Cl_6_, LiMCl_6_ (M = Ta/Nb), Li_2+*x*_Y_*x*_Zr_1-*x*_Cl_6_	~ (1–2) × 10^–3^ to ~ 1 × 10^–4^	[55, 79, 96, 121]
Wet-chemical (Solution-mediated)	Halide precursors dissolved in protic/aprotic solvents followed by controlled precipitation and annealing; enables fine control over particle size, morphology, and defect distribution	High compositional homogeneity; reduced grain-boundary resistance, improved microstructural uniformity, and enhanced interfacial stability, leading to improved cycling performance	Li_3_InCl_6_, Li_3_YCl_6_ Li_3_ScCl_6_	~ (1–2 × 10^–3^	[89, 125, 132]

### Mechanical Properties

The mechanical properties of SEs are critically important in ASSBs. Further, importantly, for HSEs, mechanical behavior characterizes a key yet often understated bottleneck that directly limits interfacial stability, dendrite suppression, and practical processability. Poor mechanical stability can cause contact at electrode/electrolyte interfaces, resulting in detachment from current collectors. This results in the formation of a void within the electrolyte and initiates crack formation in the SE under external pressure. Therefore, these effects can boost dendrite formation, cracking, and pulverization, eventually leading to breakdown in electrochemical performance [137, 138].

Elastic properties, such as the bulk and shear moduli, are important for assessing the mechanical performance of SE. The bulk modulus of Li_3_MX_6_ is considerably lower (16–22 GPa) and decreases with halide group. This value is significantly lower than that of well-known oxide-based SE, such as LLZO (∼ 100 GPa) and slightly lower than that of sulfide-based SEs (22–33 Gpa) [139–141]. The comparatively low bulk modulus of HSEs improves their ability to contact cathodes, thereby enhancing interfacial contact and reducing interfacial resistance [142, 143]. However, this relatively low stiffness also limits their ability to mechanically suppress lithium dendrite propagation, highlighting a fundamental trade-off between interfacial contact and mechanical robustness.

Along with this, ductility and deformability are important mechanical properties of SEs for practical ASSB applications. The SEs need sufficient deformability and ductility to accommodate the volume changes in electrode materials during battery charge/discharge. Thereby reducing stress buildup at the interface and maintains stable solid–solid contact. In contrast, insufficient deformability leads to stress accumulation, interfacial delamination, and progressive loss of contact during cycling.

Recently, Han et al. [55] synthesized a series of Li_2+*x*_Y_*x*_Zr_1-*x*_Cl_6_ (*x* = 0.4, 0.5, 0.6, and 0.7) HSEs via a melting method. They thoroughly examined the influence of different cooling processes on the formation and dispersion of defects within the electrolyte structure, as well as on mechanical behavior during battery cycling. They used various analytical techniques, such as synchrotron and laboratory powder X-ray diffraction (XRD), cryo-transmission electron microscopy (Cryo-TEM), and electron paramagnetic resonance (EPR), to investigate the phase and microstructure of the samples. Results indicated that the ion transport properties and phase structure of the Li_2+*x*_Y_*x*_Zr_1-*x*_Cl_6_ electrolyte remained largely unaffected by different cooling conditions. However, quenching introduced additional defects. Quenched samples exhibited higher Young’s modulus, as determined by atomic force microscopy (AFM) and nanoindentation measurements. The Williamson–Hall method was used to calculate the internal stress, revealing higher microstrain in the quenched material. Additionally, synchrotron X-ray computed tomography (CT) analysis further confirmed the impact of the two electrolytes on battery performance. A comparison of the porosity of positive electrode composite materials before and after cycling showed that quenched materials had a greater capacity to mitigate volume changes. In conclusion, the dispersed defect-based toughening strategy, implemented via quenching, yielded unique microstructures and mechanical properties in Li_2+*x*_Y_*x*_Zr_1-*x*_Cl_6_, which could contribute to the development of mechanically robust electrolytes for high-performance ASSBs. This research elucidates the underlying mechanisms governing the mechanical behavior of electrolyte materials and their impact on force–electrical coupling failure in ASSBs. These findings offer valuable insights for strategically modifying electrolytes to enhance their performance and reliability. These findings highlight that microstructural and defect engineering can be an effective strategy to tune the mechanical resilience of HSEs without significantly compromising ionic transport properties.

In ASSBs, the cathode is typically a composite of solid particles, such as cathode active material (CAM), SE and carbon additive. In a typical NMC-based cathode, after 6%-8% cycles, CAM undergo volume expansion, which can cause a loss of interfacial contact between CAM and SE, resulting in poor cycling [144]. To mitigate this issue, most studies perform cell cycling at high stack pressures, typically > 100 MPa. However, working with such pressure in a real battery system is challenging, which limits the real-world applications.

Recently, Hennequart et al. [145] investigated strategies to improve the interfacial contact between CAM and SE using low stack pressure by employing HSE, Li_3_YBr_2_Cl_4_. Using LiNi_0.6_Mn_0.2_Co_0.2_O_2_ cathode, they demonstrated stable ASSB operation at a stack pressure as low as 0.1 MPa Vs. LiIn alloy and 0.2 MPa vs. Li metal anode, with limited capacity loss. Additionally, they found that pressures below 5 MPa do not significantly hinder rate capability, retaining 70% of the C/20 capacity even at a 1 C discharge rate. These findings highlight the potential of HSEs to enable low-stack-pressure operation of ASSBs and facilitate practical applications and Li metal integration, offering important insights for developing scalable ASSBs. These results demonstrate the potential of HSEs to enable low-pressure operation; however, achieving stable long-term cycling under such conditions remains a key challenge. In addition, compared to sulfide-based electrolytes, HSEs generally exhibit poorer sinterability and limited plastic deformability, which hinder densification and scalable processing, thereby exacerbating interfacial contact issues [89, 143, 146].

Overall, these limitations highlight that mechanical behavior is a critical bottleneck in HSEs, requiring careful optimization to balance interfacial contact, dendrite suppression, and manufacturability.

### Cost of HSEs

The high cost of all inorganic SEs makes it hard to sell ASSBs. The cost of precursor materials is a major barrier to the widespread use of HSEs, despite their promising electrochemical performance. LiCl ($6 per 10 g, Sigma Aldrich), on the other hand, is less expensive than Li_2_S ($280 per 10 g, Sigma Aldrich), which is often used as a starting material for making sulfide-based SEs, and Li_2_O ($119 per 10 g, Sigma Aldrich), which is often used as a starting material for making halide-based SEs. InCl_3_ ($132 per 10 g, Sigma Aldrich) and YCl_3_ ($141 per 10 g, Sigma Aldrich), which are needed to make representative HSEs such as Li_3_InCl_6_ and Li_3_YCl_6_, remain expensive, which is a good thing. This reliance on costly metal halides significantly increases the overall material cost and limits scalability, thereby posing a major challenge for practical commercialization.

Therefore, recent research has increasingly focused on developing cost-effective HSEs with improved ionic conductivity and electrochemical performance.

Among these, Li_2_ZrCl_6_ (LZC) has been considered one of the cost-effective HSEs because of the abundance and low cost of zirconium, a central metal element, along with its scalable synthesis routes, high ionic conductivity, and strong electrochemical performance [92, 147, 148].

More recently, Li et al. [149] reported a series of Ta- and Nb-doped LZO HSEs with general formula Li_2–x_Zr_1–x_M_x_Cl_6_ (M = Ta, Nb; x = 0, 0.25, 0.375, 0.50, 0.625, 0.75, 0.875, 1.0), synthesized via the mechanochemical method. Among these compositions, Li_1.25_Zr_0.25_Ta_0.75_Cl_6_ exhibits a remarkably high ionic conductivity of up to 10.3 mS cm^−1^ at RT. Further, when employed as a catholyte in ASSBs with an NCM as cathode, it enables exceptional electrochemical performance, including high-rate capability up to 4 C and outstanding long-term cycling stability, achieving 82.5% capacity retention over 20,000 cycles at a high current rate of 4 C. These results directly demonstrate its potential to enhance key device-level performance metrics. Similarly, Fu et al. [150] recently reported a cost-effective halide Li_1.3_Fe_1.2_Cl_4_ capable of working as a cathode, catholyte and ionic conductor. This enables a high-energy density of 529.3 Wh kg^−1^ versus Li^+^/Li and delivers excellent cycling stability (90% capacity retention for 3,000 cycles at a high current rate 5 C). Furthermore, integration with nickel-rich layered oxide further increases energy density to 725.6 Wh kg^−1^, highlighting the potential for high-performance ASSBs.

While these studies demonstrate promising pathways toward reducing cost, they also highlight the need to balance material affordability with ionic conductivity and electrochemical stability.

Overall, the high cost of key halide components, combined with the need to maintain high electrochemical performance, represents a critical bottleneck that must be addressed for the practical implementation of HSE-based ASSBs.

## Interfaces in Halide-Based Solid Electrolytes for ASSBs

In ASSBs, SEs serve as both the ionic conductor and the physical separator between the cathode and the anode. Ion transport across the cathode|SE and SE|anode interfaces drive electrochemical reactions during charge and discharge cycles, with the SE usually positioned between densely packed electrode layers. Thus, ionic transport kinetics, interfacial resistance, electrochemical stability, and long-term cycling performance of ASSBs are all significantly influenced by the physicochemical characteristics and stability of these interfaces [151]. Critically, interfacial instability represents one of the most fundamental and performance-limiting bottlenecks in HSEs, as even materials with excellent bulk ionic conductivity can fail due to interfacial degradation. Despite their favorable bulk properties and benefits, HSEs face several interfacial challenges that must be addressed to achieve optimal performance [152]. Some of these are the formation of an interphase under electrochemical polarization, the loss of mechanical contact during repeated cycling, the chemical instability of lithium metal anodes, and the increased interfacial resistance caused by interdiffusion or space-charge effects at the boundaries between the electrode and the electrolyte. These degradation processes are tightly linked and often cause the impedance to increase continuously, the active contact area to shrink, and eventually, electrochemical failure, making long-term cycling stability very difficult. Many of these interfacial problems are closely related to the mechanical properties of HSEs. For example, insufficient stiffness or insufficient deformation can cause contact loss, void formation, and crack propagation during cycling.

Thus, to fully utilize the electrochemical potential of HSEs in next-generation ASSBs, systematic interface engineering techniques—such as buffer layers, surface coatings, compositional tuning, and microstructural optimization—are crucial.

These interfacial issues and their associated mitigation techniques can be roughly classified into three primary categories:

*Halide SE | Anode Interface***:** The primary problems at this interface are instability in the reduction process and the development of lithium dendrites. This interface is particularly critical, as uncontrolled reduction reactions and dendrite penetration can lead to rapid short-circuiting and catastrophic cell failure. Artificial interlayers, protective coatings, and alloying with metals that work well with lithium are some of the ways to reduce side reactions, keep the chemical and mechanical integrity, and ensure stable lithium-ion transport over long periods of cycling.
2.*Halide SE | Cathode Interface***:** At the cathode, oxidative stability, chemical compatibility, and fast lithium-ion transport are all highly significant. Interfacial degradation at high voltages can lead to parasitic reactions, interphase formation, and increased resistance, which progressively deteriorate rate capability and cycling performance. Surface modification of cathode active materials, halide SE doping, and composite cathode design improve interfacial contact, decrease resistance, and boost overall electrochemical performance, especially when the voltage is high.
*Bilayer and Dual Solid Electrolyte Strategies***:** Multilayer designs that combine SEs with different properties can improve the stability of the anode and cathode interfaces at the same time while keeping substantial ionic conductivity. Such strategies are particularly important for decoupling conflicting requirements at the two interfaces, such as reductive stability at the anode and oxidative stability at the cathode. For instance, a lithium-metal-compatible SE layer near the anode can inhibit dendrite growth, and a high-voltage-stable halide layer near the cathode can ensure oxidative stability and low interfacial resistance.

**Fig. 6:**
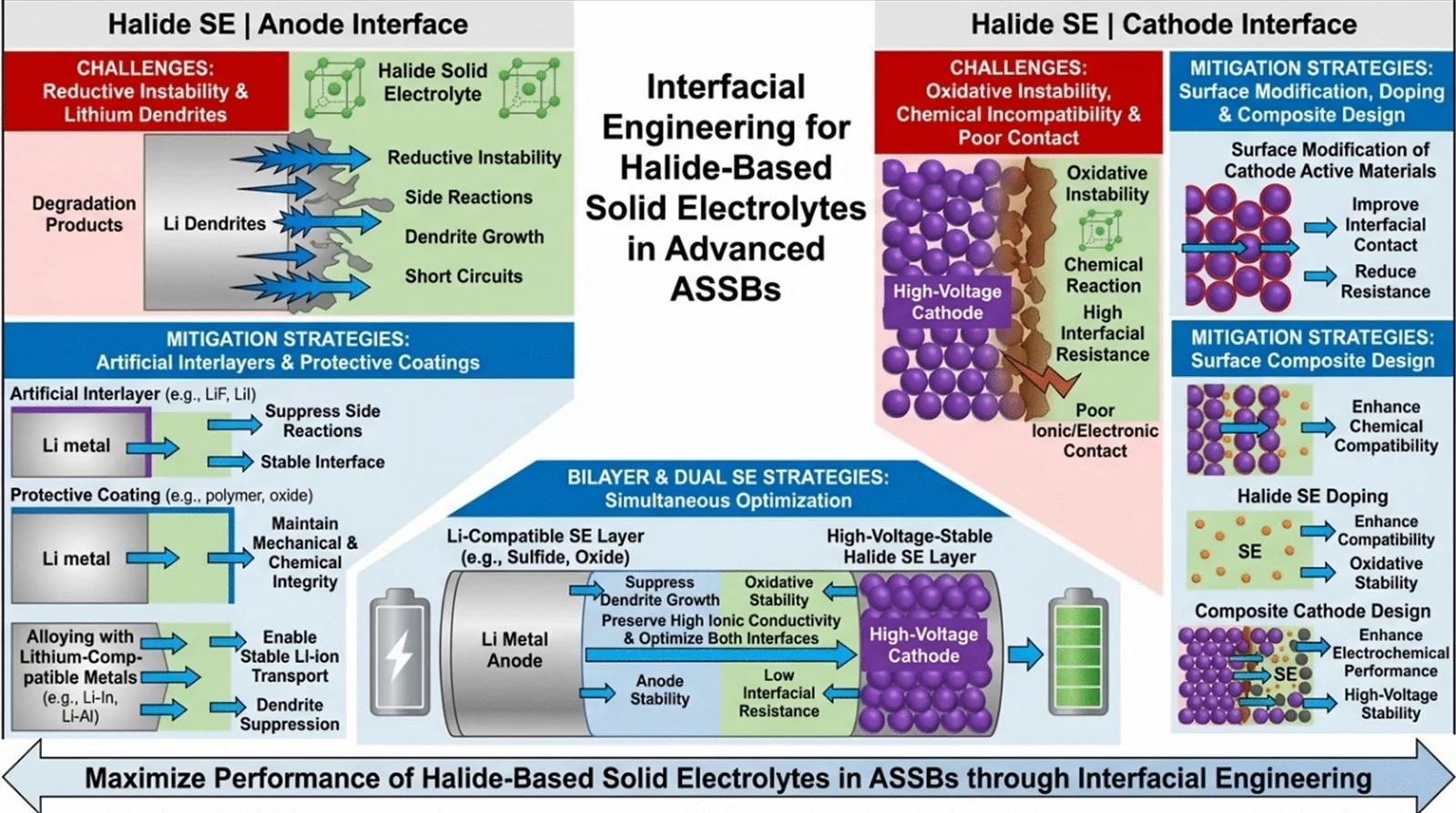
Graphical overview of interfacial challenges and mitigation strategies in HSEs for ASSBs

To fully leverage the high ionic conductivity, electrochemical stability, and energy density potential of halide-based solid electrolytes in advanced ASSBs, both anode and cathode interfaces must be carefully addressed through interlayer design, surface modification, and multilayer electrolyte strategies. Overall, the strong coupling between interfacial chemistry, transport kinetics, and mechanical integrity establishes interface engineering as a central requirement, rather than a secondary consideration, for the successful implementation of HSE-based ASSBs. This graphical abstract (Fig. 6) shows the main problems at the interface in halide-based solid electrolytes. These include reductive instability at the anode and oxidative/chemical compatibility at the cathode. It also outlines ways to address these problems, such as protective interlayers, surface modifications, and bilayer/dual-electrolyte architectures that improve electrochemical performance in ASSBs.

### Interfacial Stability at the Cathode

**Fig. 7:**
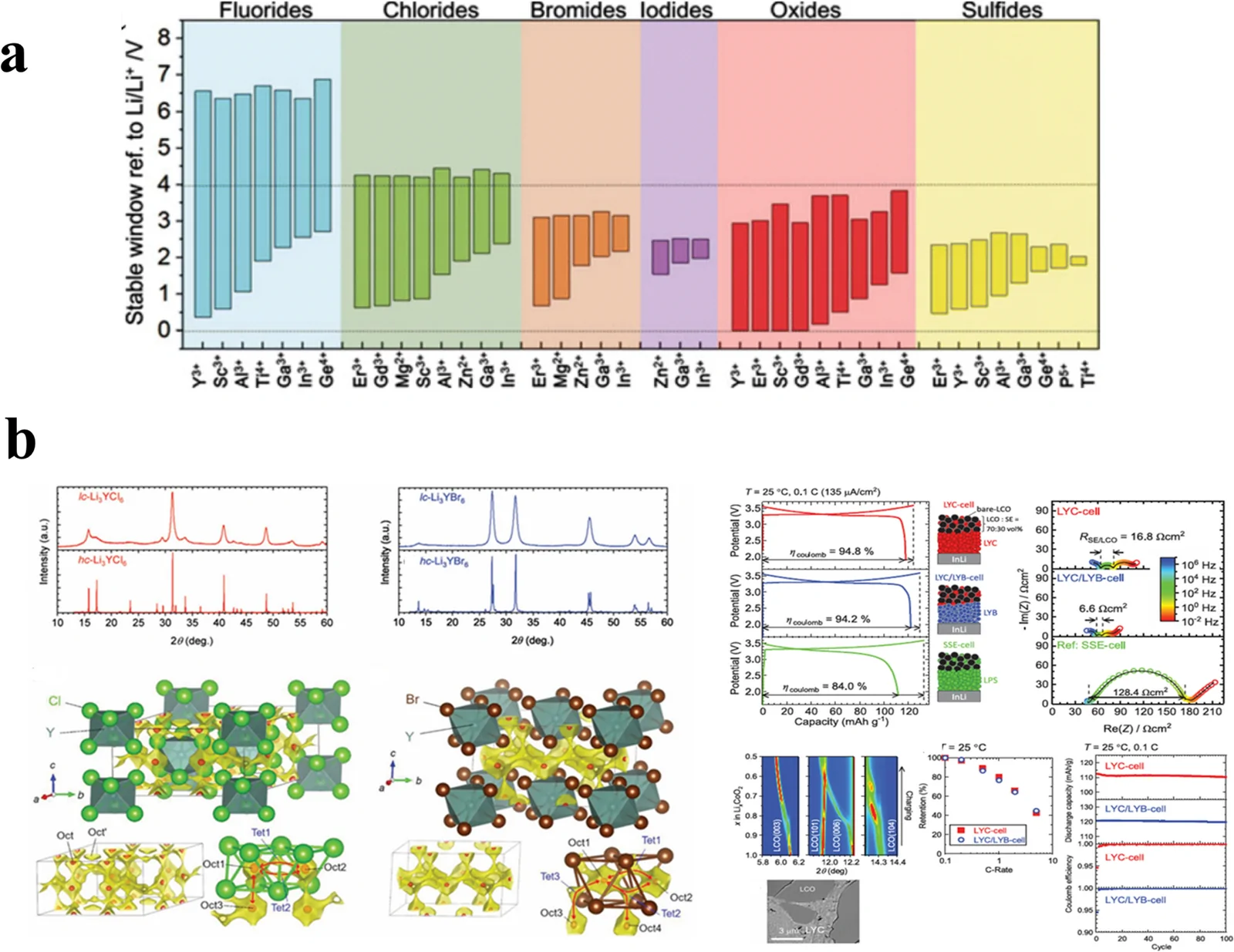
**a** Comparison of thermodynamic intrinsic electrochemical stability windows of ternary halide-, oxide-, and sulfide-based solid electrolytes. Reproduced with permission from Ref. [98]. Copyright 2019, Wiley. **b** Interfacial properties of all-solid-state lithium batteries employing halide SSEs: XRD pattern and crystal structure of synthesized Li_3_YCl_6_; initial charge–discharge curves at 25 °C and 0.1 C, in situ XRD during cycling, cross-sectional SEM image of the cathode layer, Nyquist plots after the first charge, rate performance, and cycling stability with Coulombic efficiency of LYC and LYC/LYB cells. Reproduced with permission from Ref. [89]. Copyright 2018, Wiley

The interface between the cathode and HSE is a critical determinant of overall battery performance, as it governs both oxidative stability and interfacial lithium-ion transport. Wang et al. [98] theoretically evaluated the electrochemical windows of various Li-M-X systems (M = cation, X = F, Cl, Br, I, O, S) and demonstrated that halide-based chemistries possess intrinsically wide electrochemical stability windows (Fig. 7a). Among these, fluorides exhibit the highest oxidative stability, whereas chlorides offer a favorable balance between oxidative and reductive stability. On the cathode side, chlorides show superior compatibility with high-voltage active materials compared to oxides and sulfides, effectively meeting the 4 V operational requirements of conventional lithium-ion cathodes. Bromides similarly demonstrate broad electrochemical windows and large band gaps, positioning both chloride- and bromide-based SEs as promising candidates to replace sulfide- and oxide-based electrolytes in next-generation high-energy–density batteries. Experimentally, Ansano et al. [89] reported the direct integration of Li_3_YCl_6_ SEs with high-voltage LiCoO_2_ cathodes without the need for additional protective coatings. The assembled Li_3_YCl_6_@LiCoO_2_/Li_3_YCl_6_/In-Li cell exhibited a high initial Coulombic efficiency of 94%, indicating excellent interfacial chemical and electrochemical stability under 4 V operation. Electrochemical impedance spectroscopy (EIS) analysis revealed a low interfacial resistance of 16.8 Ω cm^2^ at the Li_3_YCl_6_/cathode interface, in stark contrast to conventional Li_3_P_S4_ SEs, which showed a higher interfacial resistance of 128.4 Ω cm^2^ and a lower initial Coulombic efficiency of 84.0% when paired with LiCoO_2_ (Fig. 7b). Notably, these results primarily reflect initial cycle performance, emphasizing the need for further studies on long-term interfacial stability and evolution under repeated cycling.

### Bilayer and Dual Solid Electrolyte Strategies

HSEs exhibit excellent oxidative stability, making them well-suited for integration with high-voltage cathodes. However, their limited reductive stability renders them reactive toward lithium metal or other low-potential anodes, restricting their direct use as single-layer separators [6, 89, 153–155]. To overcome this limitation, bilayer solid electrolyte architectures have been developed. In such configurations, the halide SE interfaces with the cathode to leverage its oxidative robustness, while a sulfide- or oxide-based SE layer faces the anode to mitigate reductive instability. This design enhances interfacial compatibility and overall battery performance [75, 127, 156].

**Fig. 8:**
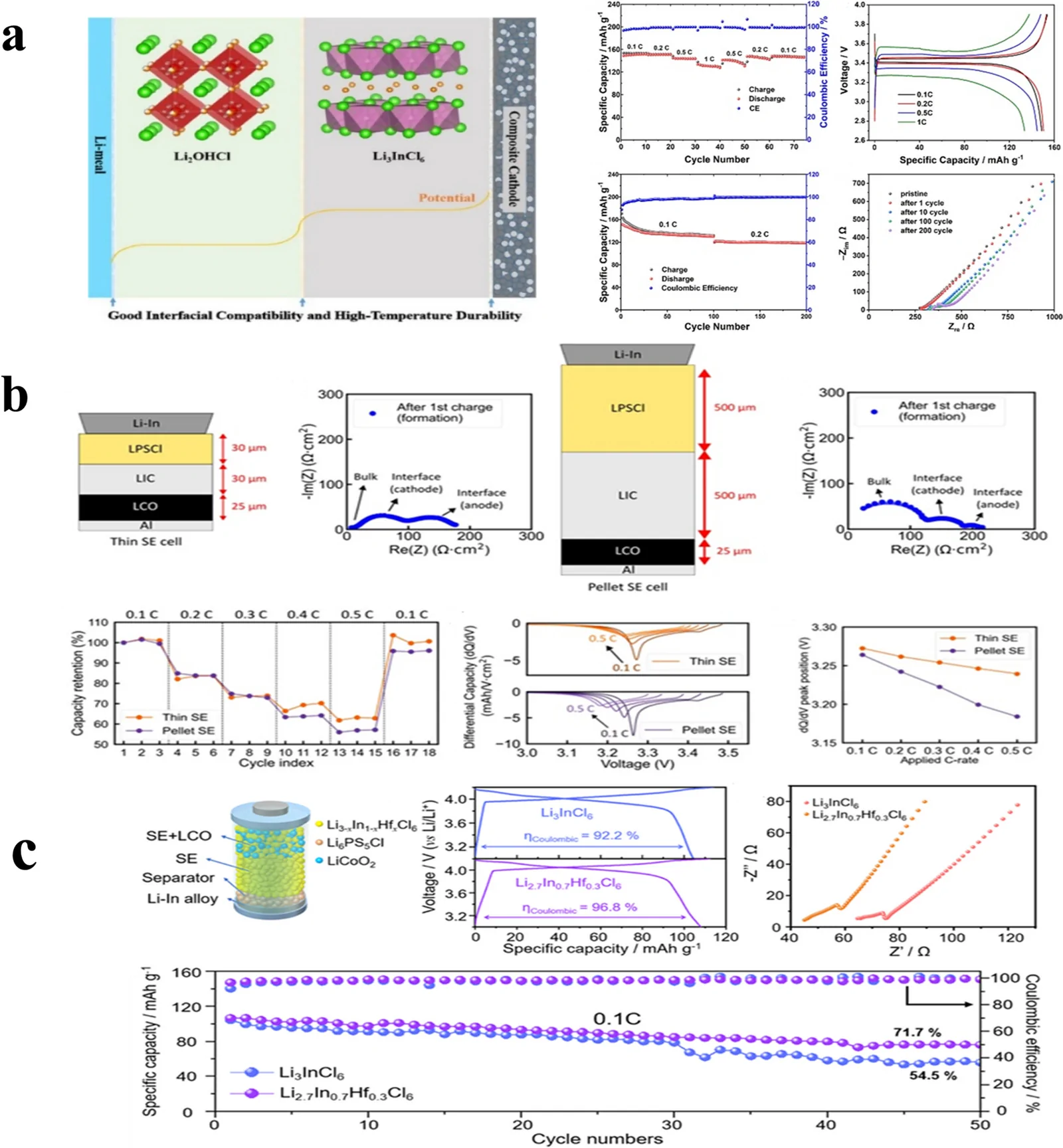
**a** Schematic illustration of a bilayer halide electrolyte interface and electrochemical performance of LiFePO_4_-Li_3_InCl_6_/Li_3_InCl_6_/Li_2_OHCl/Li batteries operated at 80 °C, including rate capability and long-term cycling behavior with corresponding interfacial resistance evolution. Reproduced with permission from Ref. [157]. Copyright 2022, American Chemical Society. **b** Schematic of an Al|LiCoO_2_|Li_3_InCl_6_|Li_6_PS_5_Cl|Li-In cell employing a thin bilayer solid electrolyte, Nyquist plot after the first charge, capacity retention at various C rates, differential capacity (dQ/dV) curves, and corresponding peak voltage shifts. Electrochemical measurements were conducted at room temperature under a stack pressure of 15 MPa. Reproduced with permission from Ref. [158]. Copyright 2024, American Chemical Society. **c** Schematic of a LiCoO_2_@Li_3–*x*_In_1–*x*_Hf_*x*_Cl_6_/Li_3–*x*_In_1–*x*_Hf_*x*_Cl_6_/Li_6_PS_5_Cl/Li-In all-solid-state lithium battery, including first-cycle charge–discharge voltage profiles, Nyquist plots after the first cycle, and cycling performance comparison at 0.1 C. Reproduced with permission from Ref. [159]. Copyright 2023, American Chemical Society

Deng et al. [157] implemented a bilayer strategy using Li_3_InCl_6_/Li_2_OHCl for LiFePO_4_-based cathodes. In their configuration (LiFePO_4_-Li_3_InCl_6_/Li_3_InCl_6_/Li_2_OHCl/Li), the halide layer faced the cathode to provide oxidative stability, while Li_2_OHCl interfaced with the lithium anode to ensure reductive stability. The resulting cells demonstrated good rate performance, delivering an initial discharge capacity of 148.8 mAh g^−1^ at 0.1 C and 133.5 mAh g^−1^ at 1 C, and maintained 120 mAh g^−1^ after 100 cycles at 0.2 C (Fig. 8a).

Similarly, Kim et al. [158] fabricated free-standing thin-film bilayer SEs composed of Li_3_InCl_6_ and Li_6_PS_5_Cl via slurry casting and lamination. When incorporated into Al|LCO|LIC|LPSCl|Li-In cells, the thin-film bilayer enhanced interfacial contact and ionic transport. Compared to thicker pellet-based SEs, these thin bilayer cells achieved ~ 10% higher rate performance at 0.5 C and exhibited less than 5% capacity fade over the first 30 cycles (Fig. 8b).

**Fig. 9:**
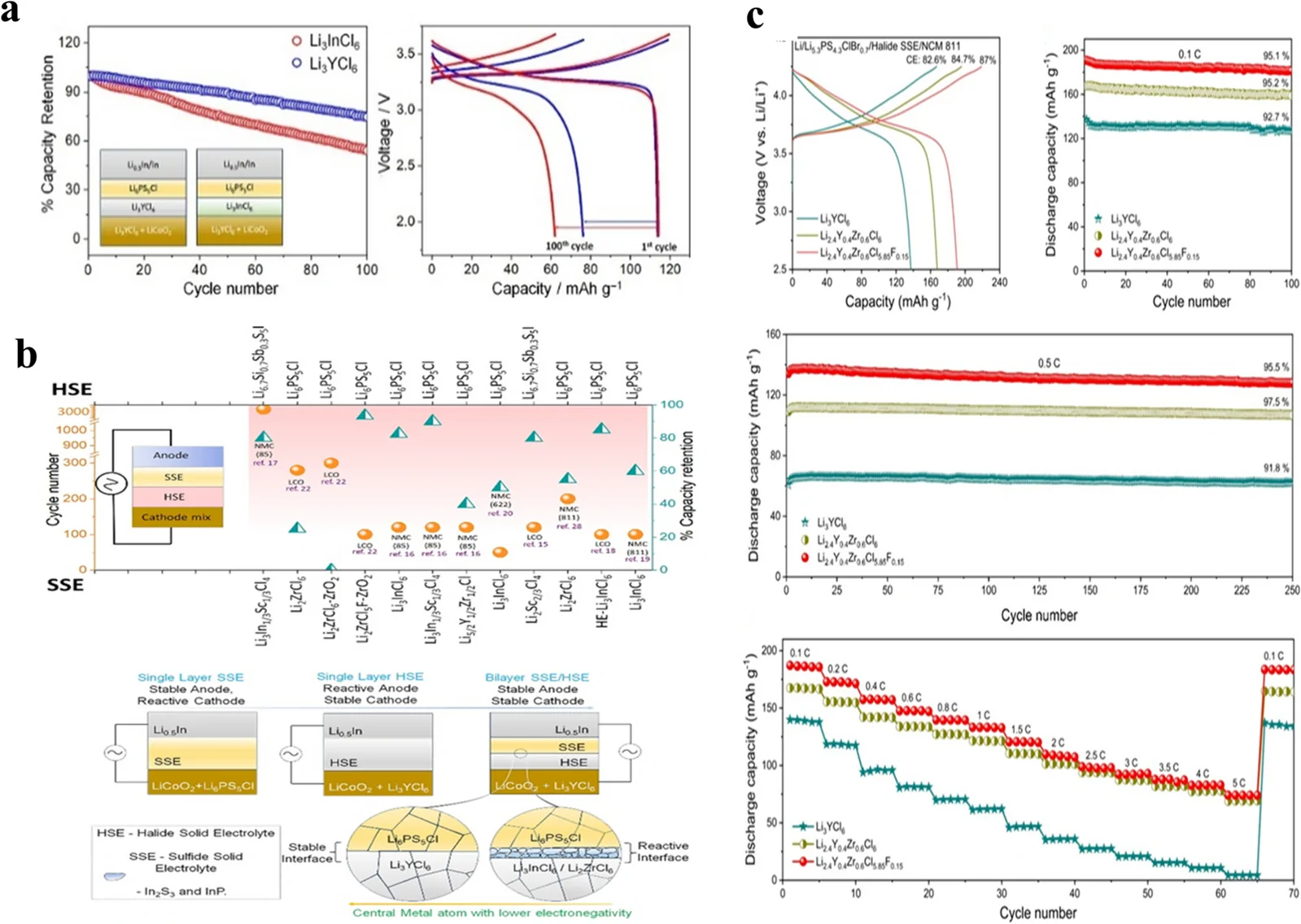
**a** Comparison of capacity retention as a function of cycle number for two bilayer cells employing different bilayer separator configurations but the same catholyte (inset: schematic illustration of the bilayer cell configuration). The corresponding 1st and 100th cycle charge–discharge profiles recorded at a C/10 rate for (blue) LiCoO_2_:Li_3_YCl_6_ | Li_3_YCl_6_ | Li_6_PS_5_Cl | Li-In and (red) LiCoO_2_:Li_3_YCl_6_ | Li_3_InCl_6_ | Li_6_PS_5_Cl | Li-In bilayer separator cells are also shown. Additionally, a literature survey summarizing reported bilayer ASSBs, including bilayer SE configurations, cycle numbers, and capacity retention, is presented. **b** A schematic comparison of monolayer and bilayer cell configurations employing two representative halide SEs, namely, Li_3_InCl_6_ and Li_3_YCl_6_ in combination with Li_6_PS_5_Cl is included to highlight their respective merits and drawbacks. Reproduced with permission from Ref. [117]. Copyright 2024, American Chemical Society. **c** Charge–discharge voltage profiles at a 0.1 C rate, cycling stability at 0.1 and 0.5 C rates, and rate capability performance of Li_3_YCl_6_, Li_2.4_Y_0.4_Zr_0.6_Cl_6_, and Li_2.4_Y_0.4_Zr_0.6_Cl_5.85_F_0.15_ solid electrolytes. Reproduced with permission from Ref. [160]. Copyright 2024, American Chemical Society

Recently, Wang et al. [159] reported a halide–sulfide bilayer electrolyte system composed of Li_2.7_In_0.7_Hf_0.3_Cl_6_ and Li_6_PS_5_Cl for ASSLBs. The Hf-substituted halide electrolyte exhibited a high room-temperature ionic conductivity of 1.28 mS cm^−1^, indicating enhanced lithium-ion transport resulting from lattice distortion and increased vacancy concentration. The authors assembled LiCoO_2_@Li_3-*x*_In_1-*x*_Hf_*x*_Cl_6_/Li_3-*x*_In_1-*x*_Hf_*x*_Cl_6_/Li_6_PS_5_Cl/Li-In cells and demonstrated superior electrochemical performance compared to cells employing pristine Li_3_InCl_6_. Notably, the bilayer system delivered a reversible capacity of 76.3 mAh g^−1^ after 50 cycles with a capacity retention of 70.8%, highlighting the effectiveness of cation substitution and bilayer electrolyte engineering in improving cycling stability and interfacial compatibility in halide-based ASSLBs (Fig. 8c). Samanta et al. [117] (Fig. 9a, b) systematically investigated the chemical compatibility of a series of halide-based solid electrolytes, including Li_3_YCl_6_, Li_3_InCl_6_, Li_3_ScCl_6_, Li_3_ErCl_6_, and Li_2_ZrCl_6_, with the sulfide electrolyte Li_6_PS_5_Cl in bilayer configurations for all-solid-state batteries. Multiple cell architectures were evaluated, namely: i) LiCoO_2_:Li_3_YCl_6_ | Li_6_PS_5_Cl | Li-In, ii) LiCoO_2_:Li_3_YCl_6_ | Li_3_InCl_6_ | Li_6_PS_5_Cl | Li-In, iii) LiCoO_2_:Li_3_YCl_6_ | Li_3_YCl_6_ | Li_6_PS_5_Cl | Li-In, iv) LiCoO_2_:Li_3_YCl_6_ | Li_3_ErCl_6_ | Li_6_PS_5_Cl | Li-In, and v) LiCoO_2_:Li_3_YCl_6_ | Li_3_ScCl_6_ | Li_6_PS_5_Cl | Li-In.

Among these, the LiCoO_2_:Li_3_YCl_6_ | Li_3_InCl_6_ | Li_6_PS_5_Cl | Li-In bilayer cell setup exhibited a capacity retention of 54.3% after 100 cycles, indicative of unfavorable interfacial reactions between Li_3_InCl_6_ and the sulfide electrolyte. In contrast, the LiCoO_2_:Li_3_YCl_6_ | Li_3_YCl_6_ | Li_6_PS_5_Cl | Li-In configuration delivered an improved capacity retention of 75% over the same cycling period. Notably, bilayer systems incorporating Li_3_ErCl_6_ | Li_6_PS_5_Cl, Li_3_ScCl_6_ | Li_6_PS_5_Cl, and Li_2_ZrCl_6_ | Li_6_PS_5_Cl, demonstrated superior interfacial stability, achieving capacity retentions of 86.7% and 49.8%, respectively, after 92 cycles at 1 C, whereas Li_2_ZrCl_6_ | Li_6_PS_5_Cl exhibited pronounced degradation with a capacity retention of 49.6%. These results clearly reveal that Li_3_InCl_6_ and Li_2_ZrCl_6_ are chemically reactive toward Li_6_PS_5_Cl, while Li_3_ScCl_6_, Li_3_ErCl_6_, and Li_3_YCl_6_ display enhanced chemical compatibility, underscoring the importance of electrolyte selection and interfacial matching in bilayer halide–sulfide electrolyte architectures. Subramanian and co-workers reported a cost-effective compositional engineering strategy for halide SEs by partially substituting low-cost Fe and Zr at the Y site and F at the Cl site in Li_3_YCl_6_ using a high-energy ball-milling approach [160] (Fig. 9c). The resulting compositions, Li_2.4_Y_0.4_Zr_0.6_Cl_6_ and Li_2.4_Y_0.4_Zr_0.6_Cl_5.85_F_0.15_, exhibited significantly enhanced room-temperature ionic conductivities of 2.05 and 1.45 mS cm^−1^, respectively, compared to pristine Li_3_YCl_6_ (0.26 mS cm^−1^). An all-solid-state battery employing Li_2.4_Y_0.6_Zr_0.4_Cl_5.85_F_0.15_ as the halide electrolyte, an NCM cathode, and Li_5.3_PS_4.3_ClBr_0.7_ argyrodite as an interlayer delivered a high initial discharge capacity of 190 mAh g^−1^ with a Coulombic efficiency of 87%, retaining 182 mAh g^−1^ after 100 cycles at 0.1 C.

**Fig. 10:**
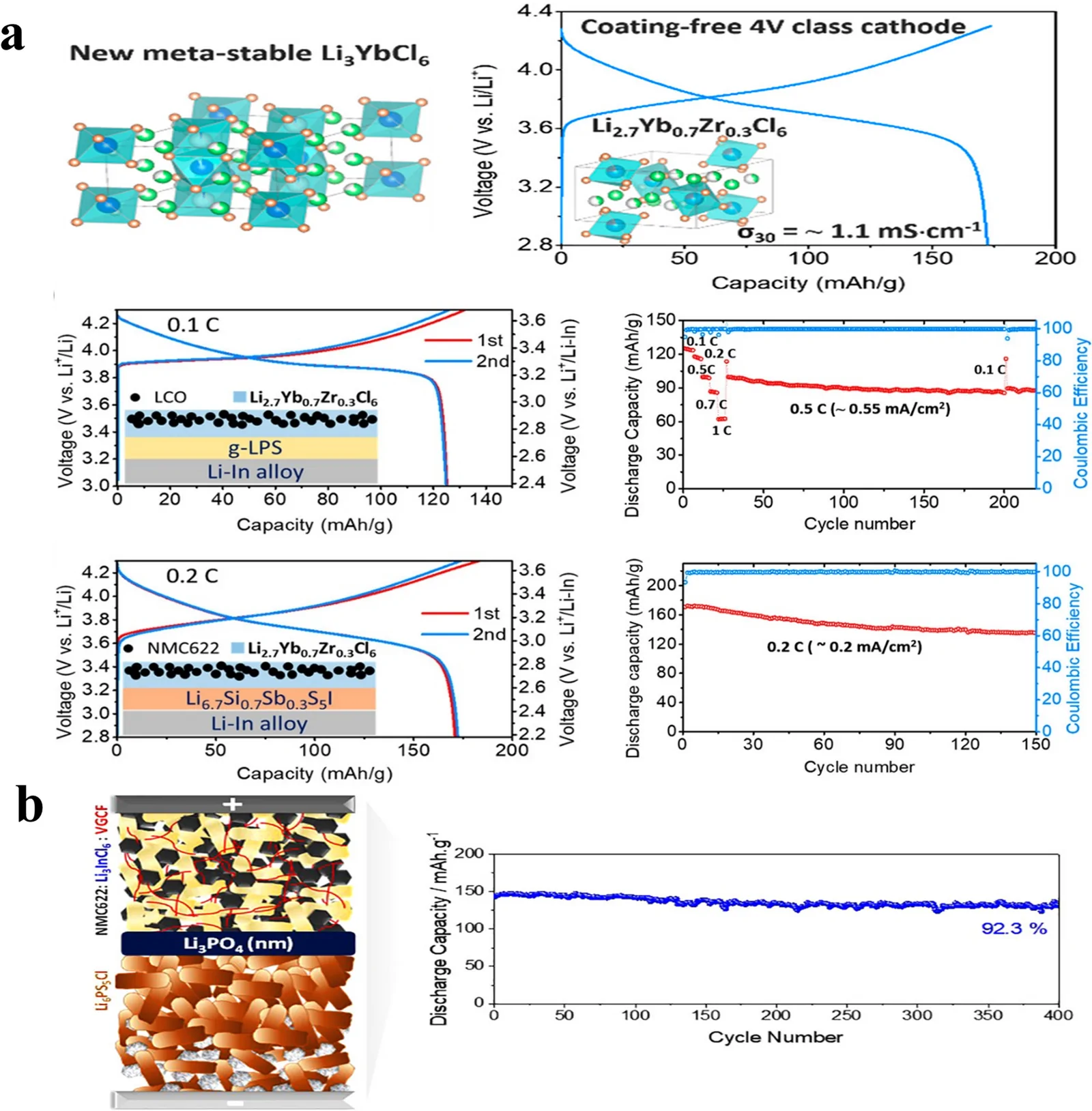
**a** Crystal structure of metastable Li_3_YCl_6_ and electrochemical performance LCO and NMC622 ASSBs employing Li_2.7_Yb_0.7_Zr_0.3_Cl_6_-350 as the solid electrolyte, cycled within voltage windows of 3.0–4.3 V and 2.8–4.3 V (vs Li/Li⁺), respectively. The corresponding charge–discharge voltage profiles from the initial two cycles of the LCO ASSB at 0.1 C and the NMC622 ASSB at 0.2 C are presented, along with the evolution of discharge capacity and Coulombic efficiency as a function of cycle number for LCO and NMC622 ASSBs. Reproduced with permission from Ref. [161]. Copyright 2021, American Chemical Society. **b** Schematic illustration of a bilayer halide electrolyte interface (NMC622:Li_3_InCl_6_:VGCF|Li_3_PO_4_-coated Li_6_PS_5_Cl|Li_6_PS_5_Cl:Li_0.5_In) and the corresponding electrochemical performance of the assembled ASSB. Reproduced with permission from Ref. [162]. Copyright 2022, American Chemical Society

Similarly, Nazar and co-workers developed Li_3_YbCl_6_ halide-based SEs and demonstrated that partial substitution of Zr on the Yb site markedly improved ionic transport [161] (Fig. 10a). While pristine Li_3_YbCl_6_ exhibited a relatively low ionic conductivity of 1.0 × 10^–4^ S cm^−1^, the aliovalently substituted Li_3−x_Yb_1−x_Zr_x_Cl_6_ achieved an enhanced conductivity of 1.1 mS cm⁻^1^. In full-cell configurations, Li_3−x_Yb_1−x_Zr_x_Cl_6_ was paired with LCO and NMC cathodes using glassy (g)-Li_3_PS_4_ and Li_6.7_Si_0.7_Sb_0.3_S_5_I interlayers, respectively. The NMC-based cells delivered an initial discharge capacity of 170 mAh g^−1^ with a high Coulombic efficiency of 97.1%, maintaining 136 mAh g⁻^1^ after 150 cycles at 0.2 C.

Despite these encouraging results, dual-electrolyte architectures commonly suffer from capacity fading during prolonged cycling due to chemical incompatibility and interfacial reactions between halide and sulfide electrolytes. To address this challenge, J. M. Tarascon group recently introduced an interfacial engineering strategy by depositing an ultrathin (1–2 nm) Li_3_PO_4_ protective layer via atomic layer deposition (ALD) at the Li_3_InCl_6_ /Li_6_PS_5_Cl interface [162] (Fig. 10b). This nanoscale interlayer effectively mitigated adverse interfacial reactions and facilitated excellent cycling stability, attaining a capacity retention of 92.3% over 400 cycles for NMC-based cathodes at an areal capacity > 3 mAh cm^−2^ in Li_3_InCl_6_-based ASSBs.

In short, dual SE configurations change the oxidative stability of HSEs on the cathode side and the reductive stability of sulfide or oxide electrolytes on the anode side. However, for long-term electrochemical stability, chemical incompatibility at electrolyte–electrolyte interfaces must be addressed. To make strong, high-performance ASSBs with HSEs, we need to continue advancing compositional modification, interfacial passivation, and nanoscale interface engineering. Table 4 shows a side-by-side comparison of common halide-based bilayer and dual solid electrolyte systems, focusing on their cell designs, interfacial strategy, and electrochemical performance.

**Table 4:** Comparison of synthesis methods for halide solid-state electrolyte

Halide SE (Cathode side)	Anode-side SE/Interlayer	Cell Configuration	Electrochemical Performance	References
Li_3_InCl_6_	Li_2_OHCl	LiFePO_4_-Li_3_InCl_6_/Li_3_InCl_6_/Li_2_OHCl/Li	1st cycle-148.8 mA h g^−1^ (0.1 C); 120 mAh g^−1^ after 100 cycles at 0.2 C	[157]
Li_3_InCl_6_	Li_6_PS_5_Cl	Al | LCO | Li₃InCl₆ / Li₆PS₅Cl | Li–In	< 5% capacity fade over 30 cycles	[158]
Li_2.7_In_0.7_Hf_0.3_Cl_6_	Li_6_PS_5_Cl	LiCoO_2_@Li_3–*x*_In_1–*x*_Hf_*x*_Cl_6_/Li_3–*x*_In_1–*x*_Hf_*x*_Cl_6_/Li_6_PS_5_Cl/Li–In ASSLB	1st cycle-108.1 mAh g^−1^ 76.3 mA h g^−1^ after 50 cycles; 70.8% retention	[159]
Li_3_YCl_6_	Li_6_PS_5_Cl	LiCoO_2_:Li_3_YCl_6_ | Li_3_YCl_6_ | Li_6_PS_5_Cl | Li–In	75% capacity retention after 100 cycles	[117]
Li₃ErCl₆	Li_6_PS_5_Cl	LiCoO_2_:Li_3_YCl_6_ | Li_3_ErCl_6_ | Li_6_PS_5_Cl | Li-In	86.7% retention after 92 cycles (1 C)	[117]
Li_3_ScCl_6_	Li_6_PS_5_Cl	LiCoO_2_:Li_3_YCl_6_ | Li_3_ScCl_6_ | Li_6_PS_5_Cl | Li-In	49.8% retention after 92 cycles	[117]
Li_2.4_Y_0.6_Zr_0.4_Cl_5.85_F_0.15_	Li_5.3_PS_4.3_ClBr_0.7_	Li/Li_5.3_PS_4.3_ClBr_0.7_/Li_2.4_Y_0.4_Zr_0.6_Cl_5.85_F_0.15_/NCM811	First cycle-190 mA h g^−1^ and 182 mAh g^−1^ after 100 cycles at 0.1 C	[160]
Li_3–*x*_Yb_1–*x*_Zr_*x*_Cl_6_	(g)-Li_3_PS_4_ and Li_6.7_Si_0.7_Sb_0.3_S_5_I	LiCoO_2_ (LCO) or LiNi_0.6_Mn_0.2_Co_0.2_O_2_ (NMC622) / Li_3–*x*_Yb_1–*x*_Zr_*x*_Cl_6_/ g)-Li_3_PS_4_ or Li_6.7_Si_0.7_Sb_0.3_S_5_I / Li-In	For NMC cathod: First cycle-170 mA h g^−1^ (CE 97.1%); 136 mA h g^−1^ after 150 cycles	[161]
Li_3_InCl_6_	Li_6_PS_5_Cl + ALD-Li_3_PO_4_	NMC | Li_3_InCl_6_ / Li_3_PO_4_ / Li_6_PS_5_Cl | Li-In	92.3% retention over 400 cycles, at an areal capacity of > 3 mAh cm^−2^	[162]

### Interfacial Stability toward the Anode

In ASSBs, Li metal is regarded as an ideal anode material owing to its exceptionally high theoretical specific capacity (3860 mAh g^−1^), the lowest redox potential (− 3.04 V vs. the standard hydrogen electrode), and low gravimetric density [63, 163–166]. Despite these advantages, the low electronegativity and strong reducing nature of Li metal render it highly reactive toward most SSEs, particularly those containing transition-metal elements, leading to severe interfacial reduction reactions upon direct contact with bare Li [167, 168].

To reduce this problem, many ASSB designs have switched from bare lithium metal to lithium–indium (Li–In) alloy anodes. This means that they lose some energy density but gain more stability at the interface. Density functional theory (DFT) calculations show that when halide SEs come into direct contact with lithium metal, they cause extensive interfacial decomposition, breaking down the electrolyte both chemically and structurally [98, 169]. An example of a reaction at the interface is:

Li_3_MX_6_ + 3Li = 6LiCl + M (M = In.,Y, etc.

**Fig. 11:**
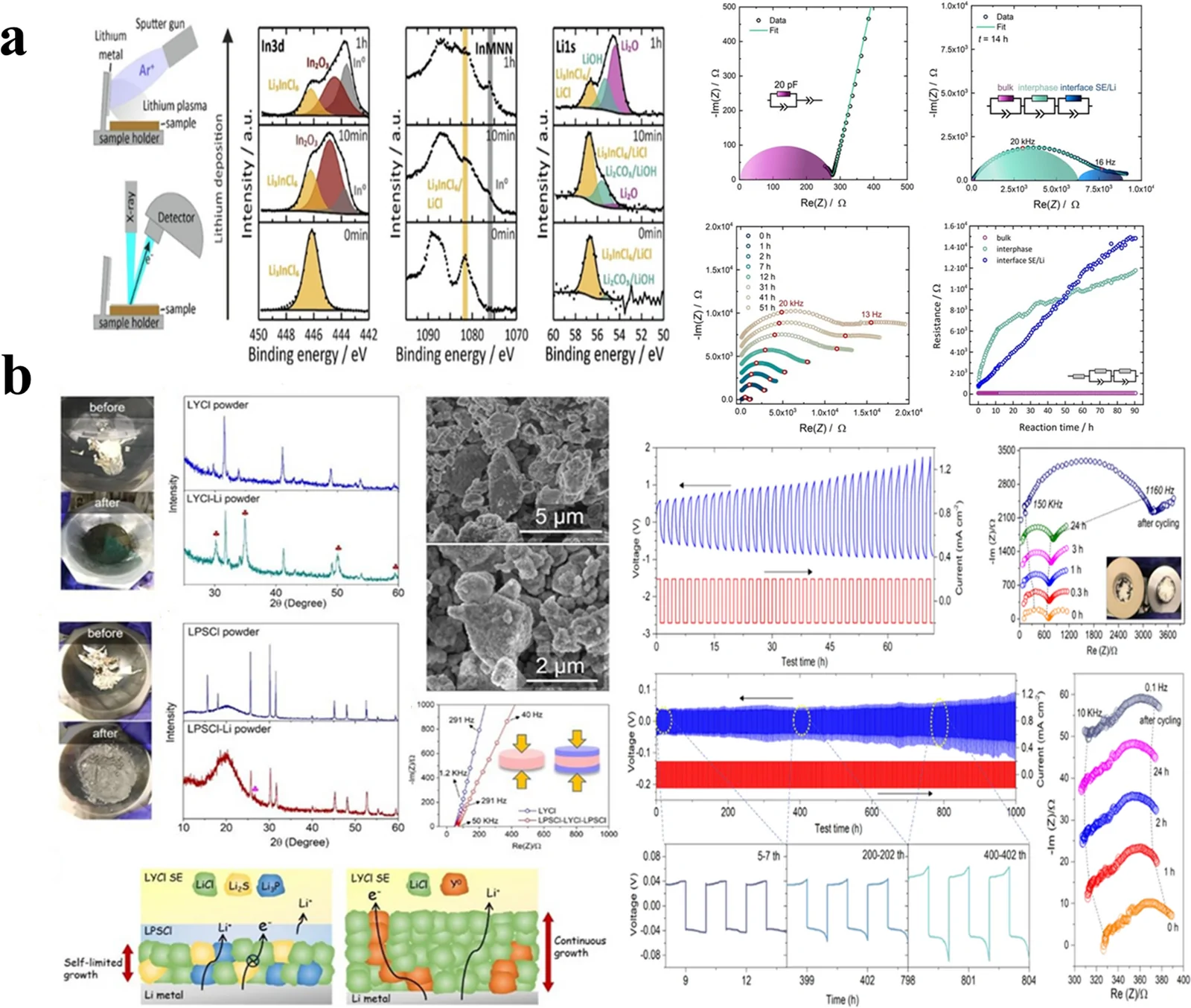
**a** Schematic illustration of Li metal deposition on HSEs using an Ar sputter gun followed by in situ X-ray photoelectron spectroscopy (XPS). Time-resolved In 3*d*, Li-1*s*, and In-MNN Auger spectra during Li deposition on Li_3_InCl_6_ reveal reductive decomposition to metallic In, which subsequently reacts with residual oxygen to form In_2_O_3_. The gradual attenuation of Li_3_InCl_6_ signals indicates progressive interphase growth. Corresponding impedance spectra of Li_3_InCl_6_ using blocking electrodes and a symmetric Li|Li_3_InCl_6_|Li cell highlight bulk ion transport, Li/SEI interfacial resistance, and the time-dependent evolution of the interphase resistance. Reproduced with permission from Ref. [175]. Copyright 2020, Wiley. **b** Photographs and XRD patterns of Li metal mixed with Li_3_YCl_6_ (LYCl) and Li_6_PS_5_Cl (LPSCl) before and after hand grinding, illustrating distinct reaction behaviors. SEM images of LPSCl and LYCl powders, room-temperature ionic conductivity of LYCl and LPSCl/LYCl/LPSCl composite pellets, and schematic designs of Li|LYCl|Li and Li|LPSCl|LYCl|LPSCl|Li cells. Voltage–current profiles and corresponding EIS spectra during rest and prolonged cycling demonstrate the improved interfacial stability enabled by the bilayer electrolyte configuration. Reproduced with permission from Ref. [170]. Copyright 2021, Royal Society of Chemistry

Ji et al. [170] used a hand-grinding method to study the reaction of halide (Li_3_YCl_6_) and sulfide (Li_6_PS_5_Cl) SEs with lithium metal at the interface. Li_3_YCl_6_ was mixed with lithium in a 3:1 molar ratio, while Li_6_PS_5_Cl was mixed with lithium in a 1:8 molar ratio to show how reactive they are with each other. After 20 min of grinding Li_3_YCl_6_ with lithium, the metallic shine of lithium disappeared, and a black powder formed. This showed that the interface was breaking down quickly. X-ray diffraction (XRD) analysis showed that crystalline LiCl was the main product of decomposition. Metallic yttrium was hard to see because it was in such a small amount and was very reactive, which caused more reactions that made Y_2_O_3_ or Y_2_(CO_3_)_2_. This quick breakdown creates a dynamically growing interface that can conduct both ions and electrons. This speeds up the degradation of the electrolyte and makes it impossible to use halide SEs directly against lithium metal without effective interfacial stabilization (Fig. 11b).

For sulfide-based SEs, the interfacial decomposition with lithium is described as:

$${\mathrm{Li}}_{{\mathrm{6}}} {\mathrm{PS}}_{{\mathrm{5}}} {\text{Cl }} + {\text{ 8Li }} \to {\text{ 5Li}}_{{\mathrm{2}}} {\text{S }} + {\text{ Li}}_{{\mathrm{3}}} {\text{P }} + {\text{ LiCl}}$$

as reported in *solid-state ionics* [171]. In contrast to halide-based electrolytes, residual metallic Li was still observable in the grayish reaction products even after 20 min of hand grinding, indicating comparatively slow interfacial reaction kinetics. Consistently, XRD analysis revealed only minor structural changes, with the emergence of a weak diffraction peak corresponding to Li_2_S, while the majority of the Li_6_PS_5_Cl phase remained intact. Among the decomposition products, Li_2_S functions as an ionic conductor with intrinsically low electronic conductivity, whereas Li_3_P exhibits relatively fast lithium-ion transport, with reported ionic conductivities on the order of ~ 10^–4^ S cm^−1^. In contrast, LiCl possesses comparatively low ionic conductivity but plays a critical interfacial stabilization role. Notably, LiCl exhibits a high interfacial energy against Li metal, which effectively suppresses lithium dendrite nucleation and propagation, analogous to the stabilizing behavior of LiF observed in previous studies [172–174]. Collectively, these results indicate that the interphase formed between Li metal and sulfide-based SEs is kinetically stable, ionically conductive, and electronically insulating, thereby enabling more benign interfacial behavior than that of halide-based SEs. This favorable interfacial chemistry supports the widespread adoption of sulfide SEs as anode-facing layers in bilayer or multilayer electrolyte architectures for ASSB**.**

To gain direct, real-time insight into the chemical evolution and reduction kinetics at the Li|halide SE interface, in situ* surface-sensitive characterization* techniques have been employed. Riegger et al. [175] (Fig. 11a) systematically investigated the interfacial reduction behavior of halide-based SEs using in situ XPS during lithium sputter deposition on Li_3_YCl_6_ and Li_3_InCl_6_ SEs. For pristine Li_3_InCl_6_, a characteristic In 3*d* peak was observed at a binding energy of 446.1 eV. Upon lithium deposition for 10 min, two additional features emerged at 444.8 and 443.7 eV, corresponding to In_2_O_3_ and metallic In^0^, respectively, while the original Li_3_InCl_6_ signal remained detectable. These observations confirm the thermodynamically favored reduction of In^3+^ to In^0^ by Li metal. Subsequently, the freshly formed indium metal readily reacts with oxygen from surface decontamination layers or residual oxygen in the measurement chamber, forming In_2_O_3_. Notably, the continued growth of the In_2_O_3_ signal after a waiting period highlights the dominant role of residual oxygen in driving secondary interfacial reactions. After approximately one hour of lithium deposition, the In 3*d* signal associated with Li_3_InCl_6_ markedly diminished, indicating progressive interfacial decomposition and the formation of a chemically evolving Li|SE interphase.

A similar reduction mechanism was observed for Li_3_YCl_6_. Within 10 min of Li deposition, Y^3+^ species were reduced to metallic Y^0^, which subsequently reacted with adventitious contaminants from the antechamber or neighboring species to form Y_2_O_3_ or Y_2_(CO_3_)_3_. These secondary reactions resulted in a pronounced broadening of the Y 3*d* XPS signal, reflecting the formation of a chemically heterogeneous interphase (Fig. 11a). Collectively, these in situ XPS results provide direct experimental evidence for the intrinsic reductive instability of halide-based SEs against Li metal and elucidate the dynamic chemical evolution of the Li|halide SE interface.

Therefore, effective strategies are required to mitigate the parasitic reactions between halide-based SEs and Li metal anodes. Interfacial modification using ionically conductive but electronically insulating layers, such as β-Li_3_N and Li_3_PO_4_ has been demonstrated to significantly enhance the interfacial stability between metal halide SEs and Li metal by suppressing continuous electrolyte decomposition and stabilizing lithium transport across the interface [162, 176]. However, the introduction of additional interlayers increases fabrication complexity and poses challenges for scalable cell manufacturing. To address these limitations, recent studies have explored fluorine-doped halide SEs, which intrinsically improve interfacial stability against Li metal by modifying the local chemical environment and reducing the thermodynamic driving force for reductive decomposition at the SE|Li interface.

**Fig. 12:**
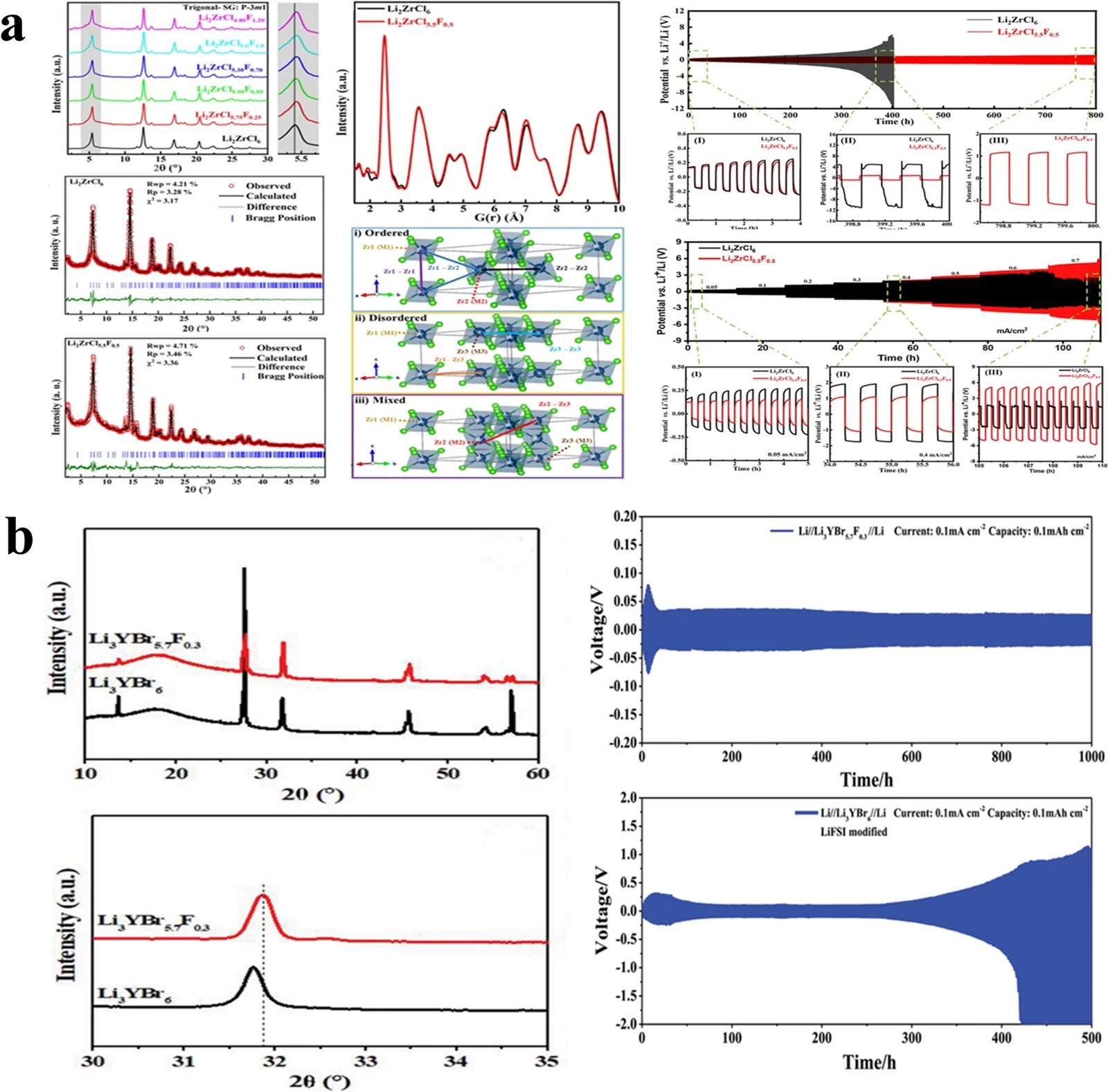
**a** Structural and electrochemical characterization of fluorine-substituted HSEs. XRD patterns and Rietveld refinements of Li_2_ZrCl_6–*x*_F_*x*_ (x = 0–1.2), synchrotron PDF analysis, and representative crystal structures illustrating ordered and disordered configurations. Lithium stripping/plating behavior of Li_2_ZrCl_6_ and Li_2_ZrCl_5.5_F_0.5_ in symmetric Li|SE|Li cells at varying current densities, demonstrating enhanced interfacial stability induced by fluorine substitution. Reproduced with permission from Ref. [119]. Copyright 2023, American Chemical Society. **b** XRD patterns of Li_3_YBr_5.7_F_0.3_ and pristine Li_3_YBr_6_ SEs. Lithium plating/stripping behavior in symmetric cells: Li‖Li_3_YBr_5.7_F_0.3_ Li and Li‖Li_3_YBr_6_‖Li (LiFSI-modified), cycled at 0.1 mA cm⁻^2^ with an areal capacity of 0.1 mAh cm⁻^2^, highlighting the improved interfacial stability induced by fluorine incorporation. Reproduced with permission from Ref. [177]. Copyright 2021, Wiley

Recently, fluorine substitution has emerged as an effective intrinsic strategy to enhance the interfacial stability of halide-based solid electrolytes against Li metal anodes. Ganesan et al. [119] (Fig. 12a) systematically investigated fluorine-substituted Li_2_ZrCl_6_ (Li_2_ZrCl_6-x_F_x_ (0 ≤ x ≤ 1.2) and correlated compositional tuning with electrochemical performance. Fluorine incorporation was found to increase the crystal density and, critically, promote the formation of a LiF-rich interphase upon contact with Li metal, as supported by DFT calculations and ex-situ XPS analyses. This LiF-containing interphase effectively suppresses continuous electrolyte decomposition and stabilizes the SE|Li interface. However, excessive fluorine substitution reduces ionic conductivity by increasing the migration barrier along the one-dimensional Li⁺ percolation pathways. An optimal trade-off between interfacial stability and ionic transport was achieved in Li_2_ZrCl_5.5_F_0.5_, where lithium stripping/plating experiments demonstrated that the stabilized interphase ultimately yields superior cycling performance compared to fluorine-free Li₂ZrCl₆, despite its slightly lower bulk ionic conductivity.

Complementary results were reported by Yu et al. [177] (Fig. 12b) who developed fluorinated Li_3_YBr_6_ (Li_3_YBr_5.7_F_0.3_) exhibiting a high ionic conductivity of 1.8 × 10^–3^ S cm^−1^. The enhanced interfacial stability was attributed to the in situ formation of a fluorine-rich interphase during Li plating and stripping, thereby enabling direct compatibility with Li metal anodes. Lithium symmetric cells with Li_3_YBr_5.7_F_0.3_ as the electrolyte cycled very stably for 1000 h at 0.75 mA cm^−2^ with an areal capacity of 0.75 mAh cm^−2^. All these studies show that fluorine substitution is a promising way to design materials that will improve both the bulk electrochemical stability and the anode interfacial compatibility of halide-based SEs. This is an excellent strategy for developing durable, effective ASSBs.

## Full-Cell Electrochemical Performance and High-Voltage Compatibility

The electrochemical performance of HSEs in high-voltage ASSBs depends on their oxidative stability, lithium-ion conductivity, and interfacial compatibility with high-energy cathodes. Unlike traditional sulfide-based solid electrolytes, which typically require protective coatings or buffer layers to prevent interfacial decomposition at voltages > 4.3 V vs. Li/Li⁺, HSEs offer broader electrochemical stability windows. This property enables direct integration with high-voltage layered oxide cathodes, such as NCM811 and LCO, eliminating the need for interfacial buffer layers. This inherent oxidative robustness renders halide SSEs highly promising for next-generation high-energy–density ASSLBs.

**Fig. 13:**
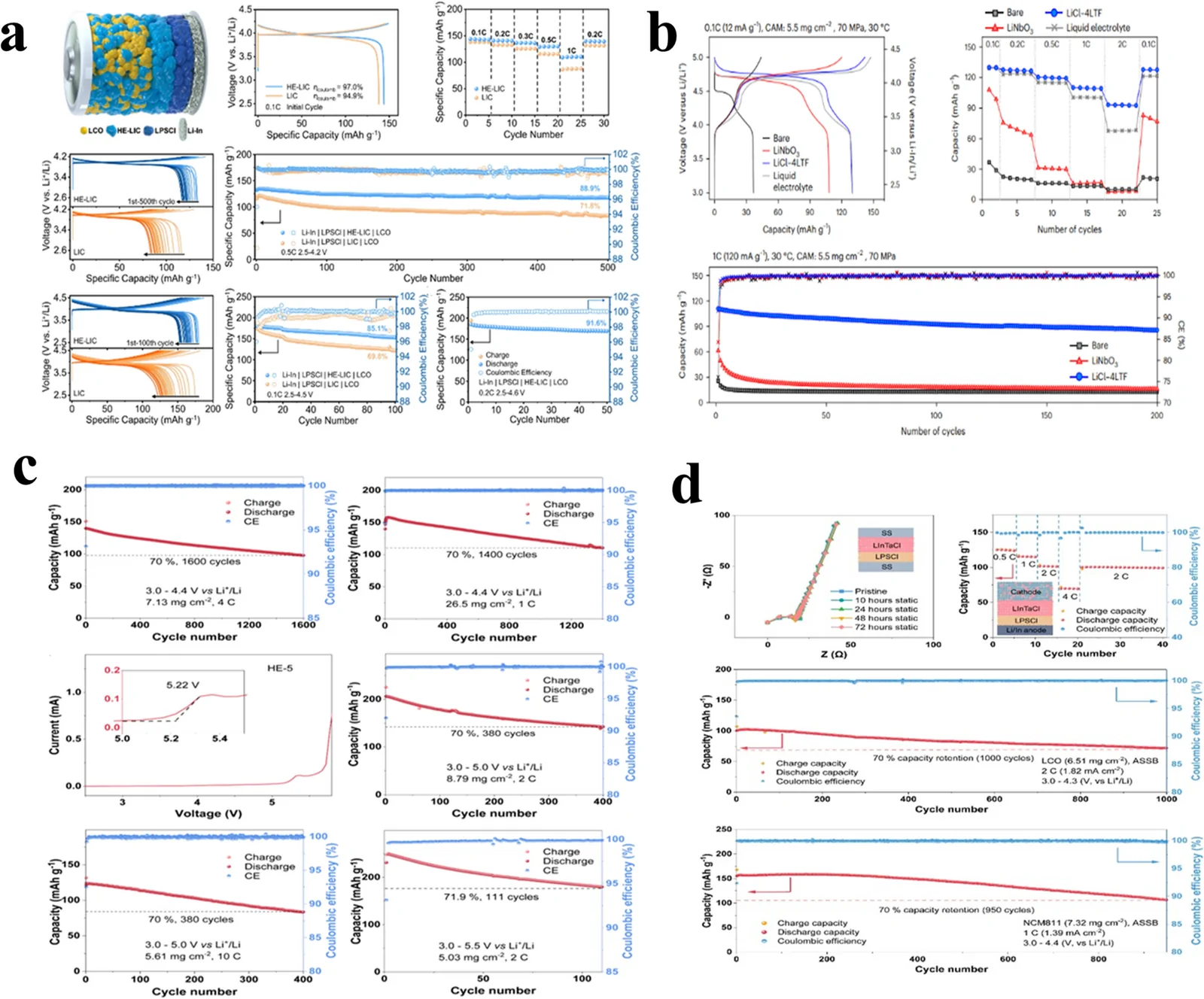
Electrochemical performance of ASSBs based on halide and fluoride solid electrolytes. **a** Cell configuration and electrochemical performance of HE-LIC cells: schematic of the cell using HE-LIC as the solid electrolyte; charge–discharge curves at 0.1 C; rate capability at 0.1, 0.2, 0.3, 0.5, and 1 C; long-term cycling at 0.5 C (2.5–4.2 V), 0.1 C (2.5–4.5 V), and 0.2 C (2.5–4.6 V). Reproduced with permission from Ref. [111]. Copyright 2024, Nature Publications. **b** Electrochemical characterization of LiCl-4Li_2_TiF_6_ with LNMO cathodes: initial charge–discharge profiles at 0.1 C (1 C = 120 mA g⁻^1^); rate capability and cycling performance with Coulombic efficiency at 1 C. Reproduced with permission from Ref. [179]. Copyright 2024, Nature Publications. **c** High-entropy halide (HE-5) ASSB performance: long-term cycling at 4 C; cycling of high-loading cathodes; LSV curves from OCP to 5.8 V (vs Li/Li⁺, 0.5 mV s⁻^1^); long-term cycling at 2 C for 5 V operation; high-rate cycling at 10 C; cycling performance and Coulombic efficiency at 2 C for 5.5 V. Reproduced with permission from Ref. [110]. Copyright 2025, American Chemical Society. **d** High-valent halide Li_2.6_In_0.8_Ta_0.2_Cl_6_ ASSBs: EIS spectra of SS//LPSCl//LiInTaCl//SS cells during 72 h static test; rate performance and Coulombic efficiency at 0.5, 1, 2, and 4 C for LCO//LInTaCl//LPSCl//Li–In; long-term cycling at 2 C for LCO and 1 C for NCM811 full cells. Reproduced with permission from Ref. [178]. Copyright © 2024, American Chemical Society

Recent studies have shown that high-entropy halide electrolytes, such as Li_2.75_Y_0.16_Er_0.16_Yb_0.16_In_0.25_Zr_0.25_Cl_6_ (HE-LIC), exhibit superior performance in Li-In | LPSCl | HE-LIC | LCO cells. The cathode was composed of LCO and HE-LIC in a 7:3 weight ratio to ensure adequate Li⁺ conduction. At 0.1 C (1 C = 140 mA g^−1^), HE-LIC cells achieved a first-cycle discharge capacity of 144 mAh g^−1^ and an initial CE of 97.0%, surpassing the performance of LiInCl_6_ cells, which delivered 138 mAh g^−1^ and 94.9%, respectively. The HE-LIC system demonstrated strong performance at high charge–discharge rates and recovered full capacity when the current was reduced. It retained 88.9% of its capacity after 500 cycles at 0.5 C, whereas the LIC system retained only 71.8%. The HE-LIC system operates effectively at high voltages due to its stability against oxidation. It maintained 85.1% of its capacity over 100 cycles at 4.5 V and 0.1 C. At 4.6 V, HE-LIC cells delivered 185 mAh g^−1^ with 91.6% capacity retention over 50 cycles and an average CE of 99.9%. Engineering improvements were implemented for each battery system. These results highlight that HE-LIC combines high Li⁺ conductivity and stability, making it a promising candidate for high-voltage ASSLBs [111] (Fig. 13a).

More recently, the high-entropy chloride Li_2.2_In_0.2_Sc_0.2_Zr_0.2_Hf_0.2_Ta_0.2_Cl_6_ (HE-5) demonstrated exceptional electrochemical performance in Li-In | Li_6_PS_5_Cl | HE-5 | NCM83 cells. Li_6_PS_5_Cl served as an interlayer to prevent anode-side decomposition of the chloride electrolyte. HE-5 exhibited high-rate cycling stability at 4 C, retaining 70% capacity over 1600 cycles with an average CE of 99.9%. High-loading cathode tests (26.5 mg cm^−2^) showed similar capacity retention over 1400 cycles. Linear sweep voltammetry revealed an oxidation onset potential of 5.22 V, significantly higher than Li_3_InCl_6_ (4.20 V), attributed to high-valence cation doping (Zr^4^⁺, Hf^4^⁺, Ta^5^⁺) that increases configurational entropy, introduces lattice distortions, and suppresses chloride oxidation. High-voltage cycling up to 5 V at 2 C maintained stable capacity over 380 cycles, and at 10 C, 70% capacity was retained over the same period. When cycled up to 5.5 V, HE-5 delivered an initial discharge capacity exceeding 230 mAh g^−1^ with stable cycling over 100 cycles [110] (Fig. 13c).

And also, recently, Ye et al. [178] (Fig. 13d) reported the superior electrochemical performance of HSEs in high-voltage ASSBs comes from their wide oxidative stability windows and good compatibility with layered oxide cathodes. Linear sweep voltammetry (LSV) studies show that high-valent and high-entropy HSEs have oxidation onset potentials above 5.0 V (vs Li/Li⁺), which is much higher than that of conventional Li_3_InCl_6_ (about 4.2 V). Improved oxidative tolerance helps high-voltage cathodes like LCO and Ni-rich NCM cycle more stably by preventing the electrolyte from decomposing prematurely. Because of this, constructed ASSBs can achieve high discharge capacities of 130–180 mAh g^−1^ at higher cutoff voltages (4.5–4.6 V), showing excellent Coulombic efficiency and long-term performance. Notably, systems based on high-valent Li_2.6_In_0.8_Ta_0.2_Cl_6_ and high-entropy chlorides maintain ~ 70% capacity retention over 1000–1600 cycles at high rates (4–10 C), and remain electrochemically stable even under 5.0–5.5 V operation, underscoring their suitability for next-generation high-energy–density ASSLBs.

Complementing halide SSEs, fluoride-based electrolytes such as LiCl-4Li_2_TiF_6_ (LiCl-4LTF) have enabled ASSBs to break traditional voltage limitations, supporting operation beyond 5 V with ultrahigh areal capacities. LiCl-4LTF-coated spinel LNMO cathodes achieved 258 mAh g^−1^ (959 Wh kg _CAM_^−1^) between 2.3–5.0 V, with 92.5% retention after 30 cycles. In pouch-type cells with 30 μm Li metal anodes, 121 mAh g^−1^ was retained over 200 cycles at 3–5 V. Ultrathick LNMO cathodes (~ 1.8 mm, 257.4 mg cm^−2^) delivered an unprecedented areal capacity of 35.3 mAh cm^−2^ (137 mAh g^−1^) at 0.77 mA cm^−2^ and 30 °C, demonstrating the exceptional synergy of high-voltage stability and fast Li⁺ transport in LiCl-4LTF. Together, halide and fluoride SSEs provide a transformative platform for high energy density, > 5 V ASSBs with both high capacity and practical scalability [179] (Fig. 13b).

## Halide-Based Solid Electrolytes in Beyond-Li-Ion Systems

The flexibility of HSEs extends post-Li-ion systems, revealing potential pathways for all-solid-state lithium–sulfur (LS-ASSBs), Li-O_2_ (LO-ASSBs), and sodium-ion batteries (Na-ASSBs). HSEs can provide several functional roles in these systems, including solid electrolytes, catholytes, or interfacial modifiers, contingent upon the battery’s chemistry. HSEs function more effectively in these systems due to their wide working electrochemical window, superior ionic conductivity, and favorable compatibility with reactive cathode chemistries at the interface [29, 56, 57, 60].

In a recent study on the LS-ASSBs, the authors assessed the compatibility of HSEs within sulfur chemistry. They used three model halides (Li_3_InCl_6_, Li_3_YCl_6_, and Li_3_YBr_6_) as composite cathode electrolytes (catholytes) to see how stable they were electrochemically and at the interface with sulfur and Li_2_S active materials. Li_3_InCl_6_ and Li_3_YCl_6_ cells degraded quickly and did not work well with sulfur at the SE/sulfur interface. On the other hand, the Li_3_YBr_6_ cell delivered improved electrochemical performance, showing a higher normalized discharge capacity of ~ 1100 mAh g^−1^ over 20 cycles. These findings highlight that HSEs can efficiently function as catholytes to control polysulfide shuttling; however, their practical implementation is constrained by interfacial instability with sulfur species and slow ionic transport within composite cathodes.

**Fig. 14:**
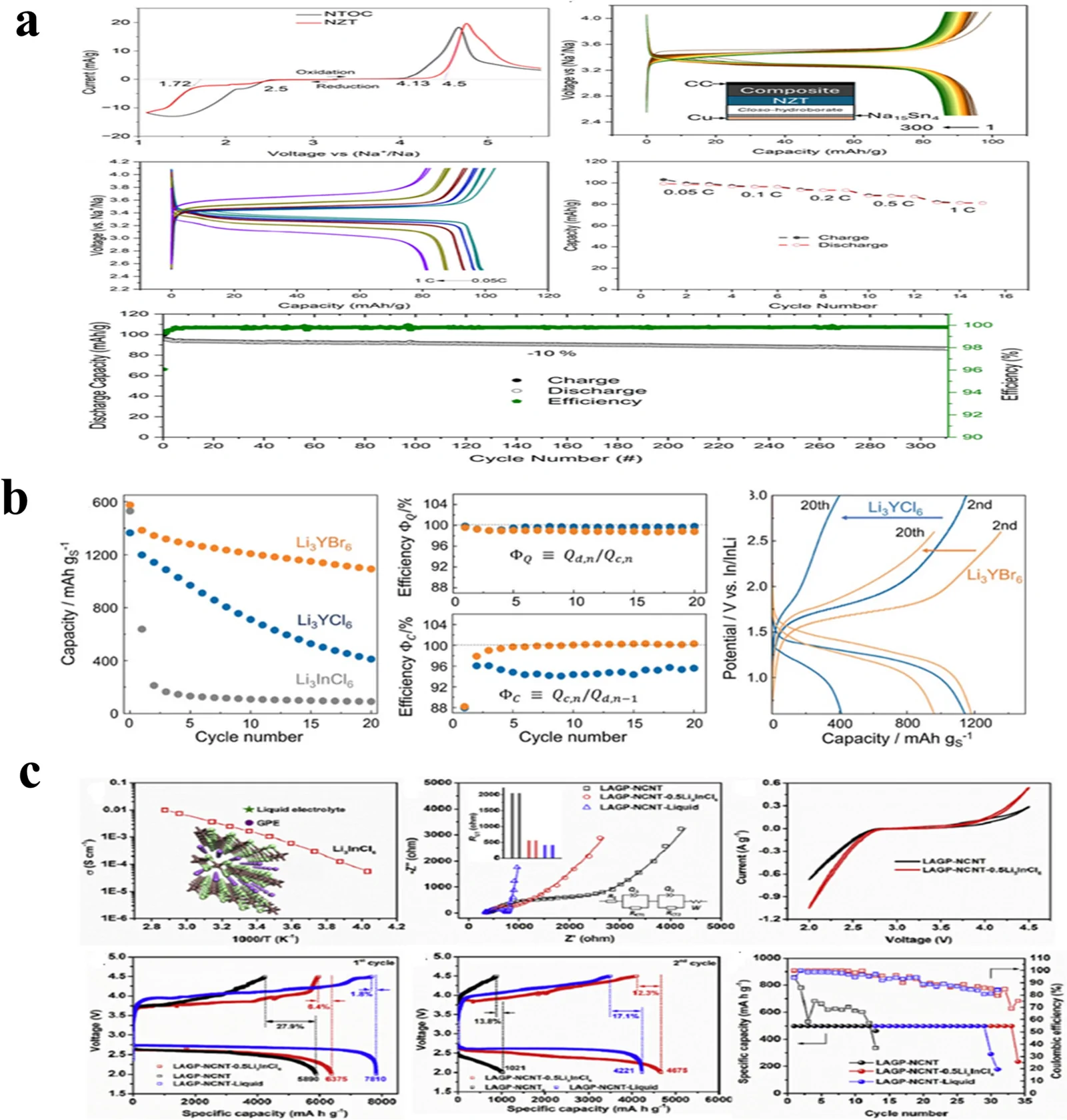
HSEs in beyond-Li-ion solid-state batteries. **a** Linear sweep voltammetry of NZT and NaTaOCl_4_ at 0.1 mV s^−1^ and electrochemical performance of NZT-based Na-ASSBs, including charge–discharge profiles at 0.2 C, rate capability (0.05–1 C), and long-term cycling at 0.2 C using Na_3_V_2_(PO_4_)_3_ cathodes and Na₁₅Sn₄ anodes. Reproduced with permission from Ref. [58]. Copyright 2025, American Chemical Society. **b** Electrochemical performance of solid-state Li–S batteries employing Li_3_InCl_6_, Li_3_YCl_6_, and Li_3_YBr_6_ electrolytes, including discharge capacity evolution, Coulombic efficiency, and representative cycling profiles. Reproduced with permission from Ref. [56]. Copyright © 2025, American Chemical Society. **c** Electrochemical characterization of halide-modified solid-state Li-O_2_ batteries, including ionic conductivity comparison, impedance spectra, charge–discharge profiles, and cycling performance of LAGP-NCNT and L_i3_InCl_6_-modified air electrodes. Reproduced with permission from Ref. [59]. Copyright © 2025, Elsevier

This study underscores essential degradation pathways in HSEs utilizing LS-ASSBs and discloses that bromide-based HSEs, encouragingly, enhance cyclability and interfacial stability relative to chloride-based SEs, providing significant insights for developing high-performance LS-ASSBs [56] (Fig. 14b).

HSEs also have distinctive benefits in LO-ASSBs, where the air cathode interfacial stability and reversibility continue to be the main challenges. Recently, Li_3_InCl_6_ has been described as an efficient interfacial modifier for a solid-state air electrode because of its high ionic conductivity, which can be prepared by a low-cost solution-based method. Integration of Li_3_InCl_6_ into the air cathode, which substantially reduced interfacial resistance, promoted reversible formation and decomposition of discharge products, and improved cycling stability. Improved Li_3_InCl_6_-modified cells delivered initial discharge capacities > 5000 mAh g^−1^ and prolonged the cycle over ~ 12 cycles (without modifier) to over 30 cycles in capacity-limited conditions. This highlights the promise of HSEs in enabling stable and efficient LO-ASSBs [59] (Fig. 14c).

Their implementation is hindered by interfacial instability in the presence of reactive discharge/charge oxygen species, restricted catalytic activity for oxygen reactions, and complications in accommodating insulating discharge products within the cell.

HSEs have recently been adapted for Na-ASSBs. Recently, a novel family of amorphous Zr-rich fluorochloride catholytes, Na_0.8_Zr_0.8_Ta_0.2_Cl_4.2-*x*_F_0.8+*x*_ (*x* = 0, 1), was synthesized through a one-step mechanochemical route to tackle the issues of ionic conductivity, oxidative stability, and mechanical deformability in Na-ASSBs. The prepared catholytes demonstrate high sodium-ion conductivity (~ 1 mS cm^−1^), low activation energy (~ 322 meV), and oxidative stability up to 4.5 V vs. Na⁺/Na, facilitated by their dual-anion (Cl^−^/F^−^) oligomeric structure. When integrated with Na_3_V_2_(PO_4_)_3_ cathodes, ASSBs delivered capacities of ~ 100 mAh g^−1^ with 90% retention over more than 300 cycles at a voltage window of 2.5–4.1 V, indicating effective mitigation of interfacial side reactions. Pairing the optimal catholytes with Na_3_V_2_(PO_4_)_2_O_2_F cathodes allowed cycling at increased voltage up to 4.4 V, hence enhancing the energy density of the battery. The amorphous Zr-rich fluorochlorides exhibited a stable electrode–electrolyte interface, underscoring their potential suitability as high-performance HSEs for high-voltage Na-ASSBs [58] (Fig. 14a).

Notwithstanding these encouraging results, significant obstacles remain, including attaining improved Na⁺ ionic conductivity comparable to Li-based systems, ensuring long-term interfacial stability with sodium metal anode, and enhancing mechanical compatibility for stable cycling.

Together, these studies show that HSEs provide a strong and flexible materials platform for high-performance ASSBs in post-Li-ion systems, including Li-O_2_, Li–S, and Na-ion chemistries.

The way these materials work and their limitations depend heavily on their chemistry. This highlights the need to design materials and engineer interfaces specifically for each battery type.

## Summary and Perspective

HSEs are an important class of inorganic solid-electrolyte materials for next-generation ASSLBs. They address a long-standing gap between the oxide- and sulfide-based electrolytes that comprise the inorganic solid-electrode class. Their unique combination of moderate-to-high ionic conductivity at room temperature, wide electrochemical stability windows, and good air stability makes them useful for high-voltage cathodes while avoiding the problems associated with traditional sulfide-based SE systems.

Among the trivalent HSEs, Li_3_InCl_6_, Li_3_ScCl_6_, and Li_3_YCl_6_ exhibit room-temperature ionic conductivities ranging from 10^–3^ to 10^–2^ S cm^−1^, supplemented by comparatively low activation energies (0.27–0.38 eV), thus enabling three-dimensional Li⁺ transport through vacancy-assisted diffusion pathways. Bromide-based trivalent HSEs offer additional benefits due to their lattice expansion affected by large-size bromide anions, which lowers migration barriers and enhances ionic conductivity. The intrinsic properties of HSEs permit them to function at high voltages (> 4.3 V) and to achieve expected energy densities of 400–500 Wh kg^−1^, when combining with high-voltage cathodes, such as nickel-rich-NMC and LCO.

The synthesis techniques have an enormous influence on the generated structural defects, microstructural uniformity, and ionic conduction in HSEs. The mechanochemical synthesis method can yield nano-sized crystalline phases with a significant disordered structural property and an increased formation of vacancies. This enables isotropic Li⁺ conduction and maintains scalability in the manufacturing process. The wet-chemical synthesis method aims for steady control over the defect distribution, morphology, and particle size. High ionic conductivities can be achieved by optimizing solvation and recrystallization pathways. Co-melting synthesis methods yield crystalline phases with different monoclinic or hexagonal structures. Synthesis has a big effect on electrochemical performance. Therefore, defect engineering and compositional optimization must be done effectively.

Interfacial stability remains a major barrier to the practical use of HSEs, despite their higher intrinsic conductivities. At the cathode interface, sulfides are more likely to oxidize than HSEs. HSE has a high coulombic efficiency and low interfacial resistance when used with high-voltage cathodes. Emphasizes the significance of strategic defect engineering and enhancing the HSE composition. Still, lithium metal anodes are unstable and degrade chemically and mechanically during cycling, so we need better interface engineering techniques.

To address these challenges, the bilayer and dual-electrolyte design, which features an HSE at the cathode and sulfide or oxide electrolytes at the anode, has been shown to be very effective at preventing parasitic reactions, slowing lithium dendrite growth, and improving long-term cycling stability.

Moreover, nanoscale interlayers and atomic-layer-deposited coatings emphasize the critical role of interfacial passivation in attaining high critical current density (CCD) and low area-specific resistance (ASR).

Beyond conventional lithium-ion batteries, halide SSEs are becoming more promising in alternative solid-state chemistries beyond regular lithium-ion batteries. In lithium–sulfur systems, HSEs function as catholytes or separators to control polysulfide migration and enhance redox kinetics. In lithium–oxygen batteries, they keep the interfaces between the electrodes and the electrolytes stable in very reactive environments. In sodium-based solid-state batteries, HSEs demonstrate the chemical adaptability of halide frameworks, although further improvements in Na⁺ conductivity and interfacial stability are required.

To speed up the process of turning halide SSEs from laboratory tests into useful energy storage devices, there are a few big problems that need to be solved. These include enhancing ionic conductivity better at low stack pressures, making the mechanical strength better against changes in electrode volume, and making sure that the chemical stability is long-term at the interfaces between the electrolyte and the electrolyte and the electrolyte and the electrode, and reducing material cost associated with rare-earth elements.

Future research should focus on the following critical directions to tackle these challenges:

(i) *High-throughput materials screening and computational design:* Employ sophisticated approaches such as density functional theory (DFT), machine learning, and data-driven screening to speed up the identification of novel halide compositions with enhanced ionic conductivity, electrochemical stability, and inexpensive elemental compositions.
(ii) *Scalable and inexpensive synthesis approaches:*The development of energy-efficient and industrially scalable synthesis methods, including continuous mechanochemical processing and solution-based approaches, is crucial for the large-scale manufacturing of HSEs with regulated microstructure and defect chemistry.
(iii) *Emphasized interface engineering and interphase regulation:* Considering that interfacial instability persists as a significant obstacle, upcoming efforts should concentrate on the development of stable interlayers, surface coatings, and multilayer electrolyte structures to mitigate parasitic reactions, decrease interfacial resistance, and improve long-term cycling stability.
(iv) *Mechanical and chemo-mechanical optimization:* Enhancing deformability, fracture resistance, and tolerance to low stack pressures is essential for ensuring stable electrode–electrolyte contact during cycling and facilitating practical cell configurations.
(v) *Balancing ionic conductivity with electrochemical stability:* Resolving the inherent trade-off between ionic conductivity and electrochemical stability by compositional modification, defect engineering, and multi-anion approaches is a critical research focus.
(vi) *Device-level validation under practical applications conditions:* Subsequent research ought to concentrate on assessments under realistic conditions (e.g., high areal loading, lower stack pressure, prolonged cycling) while reporting key metrics such as CCD, ASR, and cycle life.
(vii) Due to ongoing improvements in materials development, interface engineering, and scalable synthesis methods, HSEs are expected to play a key role in the construction of safe, long-lasting, and high-energy–density ASSBs. Achieving this goal will require a synergistic integration of fundamental materials design, interface engineering, and practical device-level optimization.
